# Chemo-radiotherapy with ^177^Lu-PLGA(RGF)-CXCR4L for the targeted treatment of colorectal cancer

**DOI:** 10.3389/fmed.2023.1191315

**Published:** 2023-06-12

**Authors:** Pedro Cruz-Nova, Brenda Gibbens-Bandala, Alejandra Ancira-Cortez, Gerardo Ramírez-Nava, Clara Santos-Cuevas, Myrna Luna-Gutiérrez, Blanca Ocampo-García

**Affiliations:** ^1^Departamento de Materiales Radiactivos, Instituto Nacional de Investigaciones Nucleares, Ocoyoacac, Estado de México, Mexico; ^2^Institute of Advanced Materials for Sustainable Manufacturing, Tecnológico de Monterrey, Mexico City, Mexico

**Keywords:** colorectal cancer, Regorafenib, PLGA nanoparticles, CXCR4L, ^177^Lu, chemoradiotherapy

## Abstract

**Introduction:**

More than 1.9 million new cases of colorectal cancer and 935,000 deaths were estimated to have occurred worldwide in 2020. Therapies for metastatic colorectal cancer include cytotoxic chemotherapy and targeted therapies in multiple lines of treatment. Nevertheless, the optimal use of these agents has not yet been resolved. Regorafenib (RGF) is an Food and Drug Administration (FDA)-authorized multikinase inhibitor indicated for patients with metastatic colorectal cancer, non-responding to priority lines of chemotherapy and immunotherapy. Nanoparticles have been used in specific applications, such as site-specific drug delivery systems, cancer therapy, and clinical bioanalytical diagnostics. C-X-C Chemokine receptor type 4 (CXCR4) is the most widely-expressed chemokine receptor in more than 23 human cancer types, including colorectal cancer. This research aimed to synthesize and preclinically evaluate a targeted nanosystem for colorectal cancer chemo-radiotherapy using RGF encapsulated in Poly(D,L-lactic-co-glycolic acid) (PLGA) nanoparticles coated with a CXCR4 ligand (CXCR4L) and ^177^Lu as a therapeutic β-emitter.

**Methods:**

Empty PLGA and PLGA(RGF) nanoparticles were prepared using the microfluidic method, followed by the DOTA and CXCR4L functionalization and nanoparticle radiolabeling with ^177^Lu. The final nanosystem gave a particle size of 280 nm with a polydispersity index of 0.347. *In vitro* and *in vivo* toxicity effects were assessed using the HCT116 colorectal cancer cell line.

**Results:**

^177^Lu-PLGA(RGF)-CXCR4L nanoparticles decreased cell viability and proliferation by inhibiting Erk and Akt phosphorylation and promoting apoptosis. Moreover, *in vivo* administration of ^177^Lu-PLGA(RGF)-CXCR4L significantly reduced tumor growth in an HCT116 colorectal cancer xenograft model. The biokinetic profile showed hepatic and renal elimination.

**Discussion:**

Data obtained in this research justify additional preclinical safety trials and the clinical evaluation of ^177^Lu-PLGA(RGF)-CXCR4L as a potential combined treatment of colorectal cancer.

## 1. Introduction

More than 1.9 million new cases of colorectal cancer and 935,000 deaths were estimated to have occurred worldwide in 2020. Colorectal neoplasia currently occupies third place in cancer incidence and second in cancer mortality. Thus, new cases and mortality rates have consistently increased with life expectancy (1). Therapies for metastatic colorectal cancer include cytotoxic chemotherapy and targeted therapies in multiple lines of treatment. Nevertheless, the optimal use of these agents has not yet been resolved (2). Regorafenib (RGF) is a small inhibitor molecule of several intracellular and membrane-bound kinases implicated in normal cell processes (intestinal absorption, epithelial proliferation) and pathological processes such as oncogenesis, angiogenesis, and the maintenance of the tumor microenvironment (3). RGF is authorized in three oncological scenarios: (a) in patients with metastatic colorectal cancer who have been unsuccessfully pretreated with fluoropyrimidine, oxaliplatin and irinotecan-based chemotherapy, anti-Vascular Endothelial Growth Factor (VEGF), and anti-Epidermal Growth Factor Receptor (EGFR) if Kirsten rat sarcoma 2 viral oncogene homolog (RAS) is not mutated; (b) in locally advanced, unresectable or metastatic gastrointestinal stromal tumors that have been previously treated with imatinib mesylate and sunitinib malate; and (c) in hepatocellular carcinoma (HCC) that has been previously treated with sorafenib (4, 5). The CORRECT, international phase III trial was performed to assess RGF in patients with metastatic colorectal cancer progressing after all approved standard therapies. The median overall survival was 6.4 months in the RGF group versus 5 months in the placebo group (*p* = 0.005). Treatment-related adverse events occurred in 93% of patients treated with RGF (3). Moreover, patients treated with targeted therapies nearly invariably develop resistance soon after initial therapy (6). Nanoparticles have been proposed to overcome many of these problems. In addition, nanoparticles have been used in specific applications such as site-specific drug delivery systems, cancer therapy, and clinical bioanalytical diagnostics and therapeutics (7–9). PLGA is one of the most useful biocompatible and biodegradable materials for synthesizing nanoparticles. The hydrolysis of PLGA leads to lactic and glycolic acid monomers, which are quickly metabolized by the body through the Krebs cycle, which result in minimal systemic PLGA toxicity and utility in drug administration (10, 11). In addition, several methods for surface modification of PLGA nanoparticles allow the particles to be targeted at organs, tissues, and tumors or the focal area, which could enable uptake increase and reduction of side effects and doses of the administered drug (12).

On the other hand, CXCR4 is the most widely-expressed chemokine receptor in more than 23 human cancers, including breast, ovarian, melanoma, prostate and colorectal cancer while expression is low or absent in many normal tissues. For example, CXCR4 expression in primary tumor cells is associated with recurrence, metastasis, and survival in colorectal cancer (13). Thus, the coating of nanoparticles with a CXCR4 ligand (CXCR4L) makes it a promising strategy for the targeted release of drugs for this type of cancer (14).

Targeted radionuclide therapy as a cancer treatment is intended for exposing malignant cells to doses of ionizing radiation, which simultaneously reduces the toxic effects of radioactivity over normal cells and the destruction of primary and metastatic tumors (15). The macrocyclic ligand 1,4,7,10-tetraazacyclodecane, *N, N′, N″, N″’*-tetraacetic acid (DOTA) is frequently employed for the preparation of metal-based radiopharmaceuticals, including ^68^Ga,^111^In, ^177^Lu, and ^90^Y (16). Lutetium-177 (^177^Lu, half-life 6.6 days) is a beta emitter (Emax 498.3 keV), which allows it to produce cytotoxicity in primary or metastatic tumors (tissue range ≈ 2 mm), and a gamma-ray emitter (E = 208 and 113 keV), which makes it useful for imaging. ^177^Lu radiopharmaceuticals based on peptides, such as [^177^Lu]Lu-DOTA-TATE, have been European Medicines Agency (EMA)- and FDA-approved in cancer patients as an excellent tool for targeted radiation therapy (17, 18).

This research aimed to synthesize and preclinically evaluate a bifunctional system of targeted chemo-radiotherapy for colorectal cancer using RGF encapsulated in PLGA nanoparticles coated with ^177^Lu and CXCR4L. The chemo-radiotherapy combination of the ^177^Lu-PLGA(RGF)-CXCR4L nanosystem is expected to improve therapeutic response in colorectal cancer models, both *in vitro* and *in vivo*, regarding single therapies.

## 2. Materials and methods

### 2.1. Materials

Regorafenib, poly(D, L-lactic acid-co-glycolic acid) [50:50 Molecular Weight (MW) 24,000–38,000 g/mol], and poly (vinyl alcohol) [PVA; Mowiol®4-88, MW 27,000] were purchased from Sigma-Aldrich (St. Louis, Missouri, United States). The DOTA [p-NH_2_-Bn-DOTA (2,2′,2″,2″‘-(2-(4-aminobenzyl)-1,4,7,10-tetraazacyclododecane-1,4,7,10-tetrayl) tetraacetic acid] was chosen as the bifunctional chelator (Macrocyclics; Dallas, TX, United States). The CXCR4L (cyclo-D-Tyr-D-[NMe]Orn[HYNIC]-Arg-Nal-Gly) was designed at ININ (Instituto Nacional de Investigaciones Nucleares, Estado de México, México) (19). The ^177^Lu was provided by ITM (Germany, EndolucinBeta, 40 GBq/mL, nca > 3,000 GBq/mg) as ^177^LuCl_3_. Dimethylformamide (DMF), diisopropylethylamine (DIPEA), 2-(1H-7-azabenzotriazol-1-yl)-1,1,3,3-tetramethyluronium hexafluorophosphate (HATU), and all other reagents were of analytical grade. The 2,3-bis-(2-methoxy-4-nitro-5-sulfophenyl)-2H-tetrazolium-5-carboxanilide; XTT) reagent was obtained from Roche Diagnostics (Indianapolis, IN, United States), and the HCT116 cell line was obtained from ATCC (Atlanta, GA, United States).

### 2.2. Synthesis of PLGA and PLGA(RGF) nanoparticles

**Figure 1:**
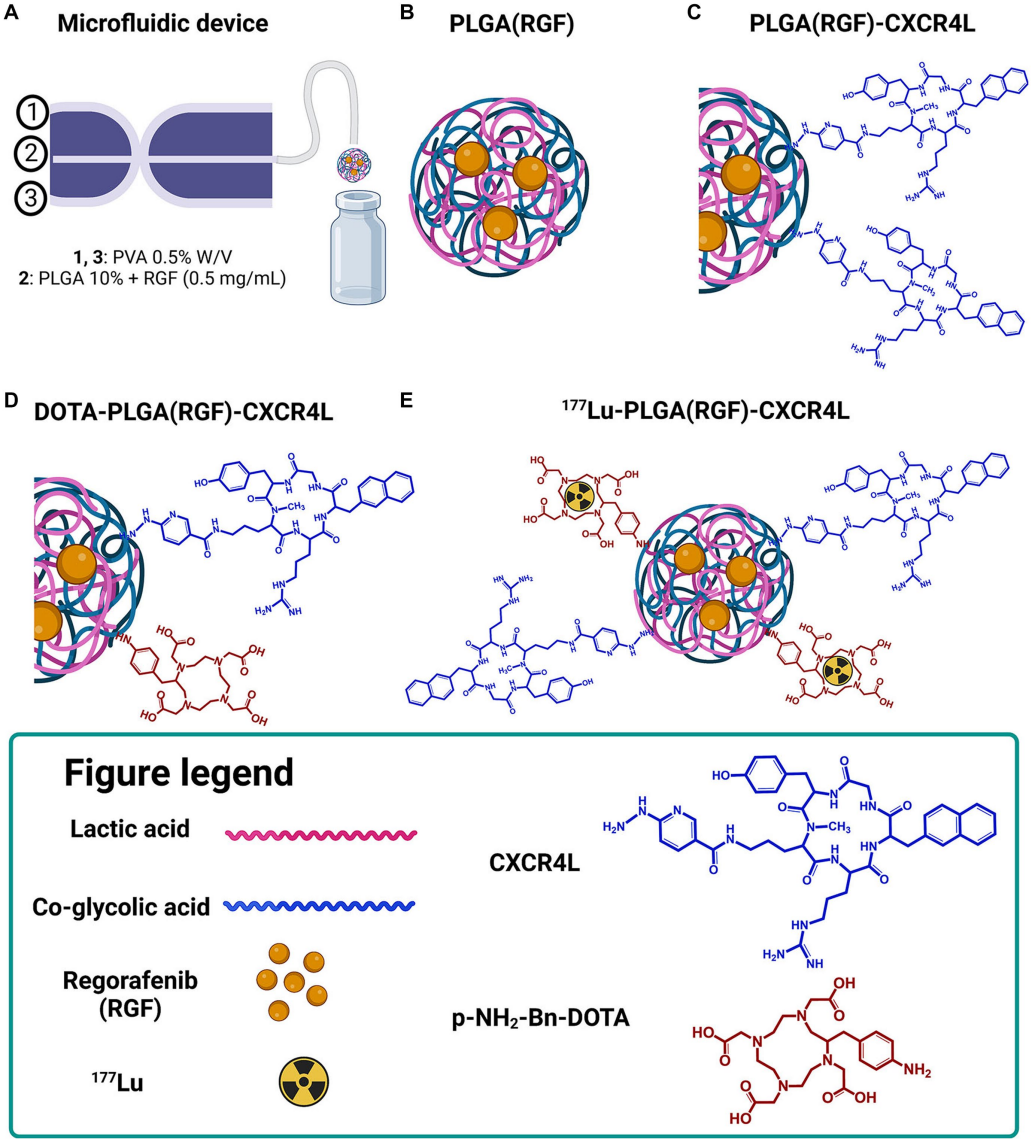
Schematic representation of the ^177^Lu-PLGA(RGF)-CXCR4L synthesis for combined therapy. **(A)** Microfluidic device (Droplet Junction Chip). **(B)** Synthesis of PLGA-entrapped RGF nanoparticles. **(C)** Functionalization of PLGA nanoparticles with CXCR4L. **(D)** Functionalization of PLGA(RGF)-CXCR4L nanoparticles with DOTA. **(E)** Radiolabeling of DOTA-PLGA(RGF)-CXCR4L with ^177^Lu.

For the synthesis of PLGA and PLGA(RGF) nanoparticles, a microfluidic system equipped with a 3D flow-focusing microfluidic device (droplet junction chip) with 100-μm channels (Dolomite Part Number: 3000158) was used (20). The first entry point was for the PVA (stabilizer surfactant) and the second entry point was for the organic phase containing PLGA in acetone (10 mg/mL) and RGF (0.5, 1.0, and 1.5 mg/mL; Figure 1A). When the fluid flow in microchannels is combined, homogeneous PLGA droplets are formed (Figure 1B).

For selecting the optimal formulation to produce stable PLGA nanoparticles loading RGF [PLGA(RGF)], Design-Expert 11.1.2.0 software was employed. A randomized factorial study without blocks was carried out with a D-optimal design and a reduced 2FI model. Thirty-six experiments were carried out, including four factors: Factor A (PVA 0.25, 0.50, and 1.00%), Factor B (RGF concentration at three levels: 0.50, 0.75, and 1.00 mg/mL), Factor C (PLGA 10 mg/mL) (17), flow rate: two levels (PLGA: 50 and 100 μL/min) and Factor D (PVA Flow Rate: 1,000 and 2,000 μL/min). Hydrodynamic size, ζ potential and polydispersity index (PDI) were measured by Dynamic Light Scattering (DLS). From these experiments, some selected nanosuspensions were evaluated for stability at room temperature for 2 weeks.

Subsequently, from optimized conditions PLGA and PLGA(RGF) nanoparticles were prepared using the droplet junction chip for providing the aqueous PVA 0.5% solution through two peripheral channels, while PLGA dissolved in acetone (10 mg/mL) was injected into the central channel of the device (Figure 1). The obtained nanosuspension was filtered by ultracentrifugation (MW Cutoff CO 30 kDa, 2,500 *g*, 30 min), freeze-dried, and stored in a B-type glass vial with an elastomer stopper and aluminum seal for further analysis. Resuspended samples with a 0.5% PVA solution (3 h, 37°C, thermo shaker) were evaluated for hydrodynamic diameter, UV–Vis, and *in vitro* and *in vivo* assays. FT-IR characterization was performed using a freeze-dried formulation.

### 2.3. Regorafenib loading evaluation

For the loading evaluation of RGF, 1 mL of acetonitrile was added to 1 mg of PLGA(RGF) and DOTA-PLGA(RGF)-CXCR4L nanoparticles and sonicated in an ultrasonic bath (Cole Parmer 9993, IL, United States, Frequency output: 42 kHz) for 30 min. The solution was filtered through a 0.22-μm membrane. The solution was injected into the HPLC system using a μBondapack C18 column (3.9 mm × 150 mm, 10 μM) as the stationary phase and methanol:acetonitrile: water (60,20,20 v/v) as the mobile phase for 10 min. Separation was carried out at a flow rate of 1 mL/min and the detection wavelength was 260 nm (21). The quantification was performed using a RGF standard curve (0.08–1.0 mg/mL, *R*^2^ = 0.95). The RGF loading capacity was calculated as the amount of RGF divided by the total weight of the nanoparticles. The RGF loading efficiency was calculated as the amount of RGF divided by the added RGF.

### 2.4. Activation of PLGA nanoparticle carboxylate groups

To activate -COOH groups of PLGA for further conjugation to the amine group of p-NH_2_-Bn-DOTA (DOTA) or hidrazyne moiety from HYNIC included in the CXCR4L structure, 10 μL of a solution containing 50 mg of the carboxylate activating agent HATU [in a basic media with DIPEA (10 μL) and DMF (200 μL)], was prepared. The reaction mixture was incubated for 15 min at 37° C and was added to the PLGA and PLGA(RGF) solutions [1 mg/mL in 0.25% PVA (v/v)].

### 2.5. Conjugation of CXCR4L and DOTA to PLGA and PLGA(RGF) nanoparticles

For the conjugation of DOTA and CXCR4L to the empty PLGA and PLGA(RGF) nanoparticles, a solution of DOTA [1 mg DOTA, 5 μL of 0.02 M HCO_3_^−^ (pH 9.0), 100 μL of 0.5% PVA (w/v)] and CXCR4L [1 mg CXCR4L in 100 μL of 0.5% PVA(w/v)] was prepared and added to the previously-activated carboxylate PLGA nanoparticle solution. The mixture was incubated at 37° C for 90 min. The nanoparticle systems DOTA-PLGA, DOTA-PLGA(RGF), PLGA-CXCR4L, PLGA(RGF)-CXCR4L, DOTA-PLGA-CXCR4L, and DOTA-PLGA(RGF)-CXCR4L were purified by ultracentrifugation (Ultra Centrifugal filters, MWCO 30,000 Da; Millipore; 16,000 *g*) until neutral pH was achieved. Finally, each filtrate was resuspended in PVA (0.5% w/v), lyophilized, and stored for further use.

### 2.6. Nanoparticles radiolabeling

The bifunctional chelator DOTA was the radiolabeling site for the different PLGA nanoparticles using acidic conditions for the ^177^Lu complexation reaction. In brief, 9.25 MBq (50 μL) of ^177^LuCl_3_ in 1 M acetate buffer (pH 5.0) was added to each PLGA nanoparticle system: DOTA-PLGA(RGF)-CXCR4L, DOTA-PLGA(RGF), DOTA-PLGA-CXCR4L, and DOTA-PLGA (1 mg/mL), and incubated for 1 h at 37° C. After radiolabeling, the nanoparticles were purified by ultracentrifugation (Ultra Centrifugal Filters, MWCO 3,000 Da; Millipore; 2,500 *g*). Both fractions (filtered and unfiltered) were measured in a dose calibrator (Capintec, United States) to calculate the radiolabeling yield. The radiochemical purity of PLGA nanoparticles was evaluated by size-exclusion high-performance liquid chromatography (HPLC) and Instant Thin Layer Chromatography with Silica Gel (ITLC-SG), as further described.

### 2.7. Dynamic light scattering and ζ potential

The particle size distribution and ζ potential analyses were carried out via Dynamic Light Scattering (DLS), using a Nanotrac analyzer (Microtrac; FL, United States). The studies were performed in an aqueous solution with a wavelength of 657 nm (20°C, current of 15.79 mA, an electric field of 14.35 V/cm, and a sampling time of 128 μs). The mean diameter and standard deviation for each sample were reported (*n* = 3). In addition, ζ potential was measured in diluted samples to ensure adequate and constant ionic strength.

### 2.8. Transmission electron microscopy

The morphological features of PLGA nanoparticles were observed by TEM in a JEOL JEM 2010 HT instrument (JEOL; Japan), operating at 200 kV. A drop of the samples was placed onto a carbon-coated copper grid and evaporated under a vacuum before measurements.

### 2.9. FT-IR spectroscopy

IR spectra of raw materials and lyophilized samples were obtained on a Perkin Elmer System 2000 spectrometer with an ATR platform (Pike Technologies), by applying attenuated total reflection, Fourier Transform Infrared (ATR-FT-IR) spectroscopy. The spectra were acquired with 50 scans in an operating range of 4,000–400 cm^−1^ and a resolution of 0.4 cm^−1^.

### 2.10. UV-Vis spectroscopy

Absorption spectra were obtained with a Perkin Elmer Lambda Bio spectrometer, in the 200–400 nm range, using a 1-cm quartz cuvette. In addition, the nanoparticle suspensions were measured by UV–Vis spectroscopy to monitor the conjugation reactions.

### 2.11. *In vitro* RGF release kinetics

For the determination of the release profile, 1 mg of DOTA-PLGA(RGF)-CXCR4L was dispersed in 1 mL of phosphate-buffered saline (PBS), within a dialysis bag (MWCO 10,000 Da). The closed bag was placed in a tube containing 10 mL of PBS as the releasing medium. Two pH media were used, simulating the physiological conditions and the intratumoral microenvironment (pH 7.4 and 5.3, respectively). The tube was maintained under slow stirring at 37°C. Aliquots of 500 μL were collected at different time points (30 min, 1, 2, 4, 24, 48, and 120 h) and the volume was replaced with fresh PBS for further correction. Reversed-phase HPLC quantified the release of RGF based on the previously-described methodology (17).

### 2.12. Nanoparticle stability

The lyophilized nanoparticles were reconstituted in PVA (0.5% w/v, 1 mL, 37°C, 3 h in thermo-shaker). Once reconstituted, the content of a vial solution (*n* = 3) was kept at room temperature at different time points (1, 24, 48, 120, and 168 h). The particle size distribution and ζ potential were measured.

Several vials containing 1 mg/mL of DOTA-PLGA(RGF)-CXCR4L were stored at 4°C for 1 year to evaluate long-term stability. The particle size distribution and ζ potential were measured.

### 2.13. Radiochemical purity

Previous to the size exclusion-HPLC analysis, the approximate DOTA-PLGA(RGF)-CXCR4L molecular weight was determined using polyacrylamide gel electrophoresis comparing with MW marker ~7–245 kDa BLUEstain™ (Cat. P007. GoldBio, United States; data not shown), Then, a YMC-Pack Diol-60 HPLC-column was used. Radiochemical purity was also performed via instant thin-layer chromatography-silica gel (ITLC-SG), using a radio TLC Scanner (mini Gita TLC, Elysia-Raytest GmbH, Angleur, Belgium) and the GINA star TLC software coupled to the equipment. The mobile phase employed in this system was MeOH:ACN:H_2_O (60:20:20) for nanosystems (Rf = 0.8) and NaCl (0.09%): 0.02 M HCl, suitable for identification of free ^177^Lu (Rf = 1). Radiochemical purity was confirmed using the size-exclusion-HPLC method, employing a photodiode array detector (Waters™ 996) with Millenium software, connected to a radio-HPLC Detector γ.RAM Model 3 (IN/US Systems). Samples were injected into the YMC-Pack Diol-60 (300 mm × 8.0 mm I.D., 5 μM, 6 nm) column using MeOH:ACN:H_2_O (60:20:20) as the mobile phase (14).

### 2.14. Cell uptake and internalization

To assess ^177^Lu-PLGA(RGF)-CXCR4L and ^177^Lu-PLGA(RGF) cell uptake, a solution containing 1 × 10^6^ HCT116 cells diluted in 1 mL of fresh medium was added to a glass tube and incubated with 10 μL (3.3 MBq) of ^177^Lu-PLGA(RGF)-CXCR4L and ^177^Lu-PLGA(RGF) at 37°C for 30 min, 1, 3, 5, and 24 h (in triplicate). Subsequently, cells were washed twice with PBS solution and counted in a gamma counter. The internalized fraction of conjugates was calculated after eliminating the membrane-bound radioactivity by incubating cells with 1 mL of CH_3_COOH/NaCl (0.2 M/0.5 M) for 2 min, washing them with PBS and counting the cell pellet. The initial activity of each treatment was considered to be 100%.

### 2.15. Cell viability

Cell viability was assessed in HCT116 cells using the XTT (2,3-bis [2-Methoxy-4-nitro-5-sulfophenyl]-2H-tetrazolium-5-carboxyanilide inner salt; 0.1 mg/mL) assay kit (Roche, Germany). Briefly, cells were seeded in 96-well culture plates (10,000 cells/well) and incubated for 24 h to allow adherence. Previous studies have demonstrated that RGF-resistant cell lines have RGF half-maximal inhibitory concentration (IC_50_) for non-resistant and resistant cells of 13.5 ± 1.5 and 55.1 ± 0.8 μM, respectively (22). Therefore, it was decided to use half the IC_50_ concentration of the RGF-resistant cells. Afterward, the cells were incubated with 50 μL (3 Bq/cell, 7.26 μM of RGF) of each treatment as follows: (1) Control (without treatment), (2) PLGA, (3) PLGA-CXCR4L, (4) PLGA(RGF), (5) PLGA(RGF)-CXCR4L, (6) RGF, (7) ^177^Lu-PLGA-CXCR4L, and (8) ^177^Lu-PLGA(RGF)-CXCR4L. The viability was evaluated at 24 h after treatment exposure. The spectrophotometric measurement of each well at 450 nm in a microplate absorbance reader (EpochTM; BioTek Instruments; United States) was performed to determine cell viability. The results were evaluated in triplicate. The absorbance from untreated cells was considered 100% of viability.

### 2.16. Clonogenic assay

HCT116 cells were used to evaluate the ability of a single cell to grow into a colony after exposure to a specific treatment. Cells (400–500 cells) were seeded in each well (six-well plate) and treated with 3 Bq/cell (RGF 7.26 μM) of (1) Control (without treatment), (2) PLGA, (3) PLGA-CXCR4L, (4) PLGA(RGF), (5) PLGA(RGF)-CXCR4L, (6) RGF, (7) ^177^Lu-PLGA-CXCR4L, and (8) ^177^Lu-PLGA(RGF)-CXCR4L dissolved in 1.5 mL of growth medium. The controls were comprised of 400–500 cells exposed to the growth medium for 48 h. After exposure, the treatments or medium were removed and replaced with fresh medium. The plates were incubated for 15 days. Surviving colonies were fixed for 10 min with 4% paraformaldehyde at room temperature and washed with type 1 water. Afterward, they were stained with 0.1% crystal violet for 30 min, washed with purified water, dried, and photographed. For quantification, 10% acetic acid was added for 5 min to extract the crystal violet, and the absorbance was measured at 590 nm in a microplate absorbance reader (EpochTM; BioTek Instruments; United States).

### 2.17. Western blot

Cell samples were transferred to a microcentrifuge tube and lysed using Tissue Cell Lysis Buffer (CAT No: GB-181-100). After centrifugation at 20,000 × *g* for 15 min and protein quantification, SDS-PAGE was carried out using 30 μg of protein in each sample. Next, proteins were transferred onto PVDF membranes (Merck Millipore) and blocked for 1 h at room temperature using PBS containing 5% bovine serum albumin (BSA). Antibodies used for western blotting were total pERK1/2 (Thr202/Tyr204; D13.14.4E; Cell Signaling-4370 1:1,000), pAkt (Ser-473; D9E; Cell Signaling-4060 1:500), and GAPDH (GeneTex, 1:5,000). Primary antibodies were incubated overnight at 4°C and washed off. Species-specific HRP-conjugated secondary antibodies were then incubated for 1 h at room temperature and washed extensively. Finally, membranes were incubated with Super Signal West Femto substrate (Thermo Fisher Scientific), and signals were detected using the *in vivo* Xtreme imaging system (Bruker, Billerica, MA, United States).

### 2.18. Apoptosis

Apoptosis in the HCT116 cell line was detected with the Muse™ Caspase-3/7 kit, Cat. No. MCH100108 (Merck Millipore; Burlington, MA, United States). Briefly, cells were seeded in cell culture flasks at a density of 1 × 10^6^ cells and exposed to 7.26 μM of RGF via the PLGA(RGF)-CXCR4L, PLGA(RGF), PLGA-CXCR4L, and PLGA nanosystems and free RGF for 24 h. Untreated cells were used as the control group. First, cells were trypsinized and suspended in 50 μL of 1X Assay Buffer BA (1 × 10^6^ cells/mL). Then 5 μL of Caspase-3/7 reagent working solution was added to each cell suspension. The suspension was then gently vortexed and incubated at 37°C with 5% CO_2_ for 30 min. After incubation, 150 μL of Caspase 7-AAD working solution was added to each tube. Finally, tubes were incubated for 5 min, protected from light. Fluorescent intensities were examined by flow cytometry using a Muse Cell Analyzer (Merck Millipore; Burlington, MA, United States) and were conducted in triplicate.

### 2.19. Biodistribution

The ^177^Lu-PLGA(RGF)-CXCR4L nanoparticle system was injected intravenously into the healthy BALB/c mice (100 μL, 2.4 MBq). The mice (*n* = 3) were sacrificed at 1, 3, 24, 48, and 144 h post-injection. The lungs, liver, kidneys, and spleen were rinsed and placed into pre-weighed plastic test tubes. The activity was determined in a well-type scintillation detector (Canberra), along with six 0.5 mL aliquots of the diluted standard, representing 100% of the injected activity. Mean activities were used to obtain the percentage of injected dose (%ID) at different times.

### 2.20. Biokinetic model and radiation absorbed dose estimation

The mean percentage of injected activity at 1, 3, 24, 48, and 144 h (%ID, *n* = 3 for each time point) of the liver, kidney, lung, and spleen were introduced into the Olinda/EXM computer code to obtain the ^177^Lu-PLGA(RGF)-CXCR4L time activity versus time function [A_h_ (*t*)], corrected by decay (λ_e_ = λ_B_ + λ_R_), for each organ (Equation 1).


(1) $$A_{h}\left(t\right)=Be^{-\left(\lambda_{eb}\right)t}+Ce^{-\left(\lambda_{ec}\right)t}$$

The integral of each time-activity curve represents the total number of nuclear transformations (N, MBq.h/MBq) of ^177^Lu in the source regions (Equation 2).


(2) $$N_{source}=\overset{\infty }{\underset{0}{\int }}A_{h}\left(t\right)dt$$

The absorbed doses delivered by ^177^Lu-PLGA(RGF)-CXCR4L to selected organs were estimated based on the following general equation (3):


(3) $$D_{target\leftarrow source}=N_{source}\;X\;DF_{target\leftarrow source}$$

D_target ← source_ is the mean absorbed dose to the target organ from a source organ and DF_target ← source_ is a dose factor that considers the fraction of absorbed energy for each nuclear emission of Lutetium-177 in the different geometries and chemical compositions of the organs. Lutetium-177 DF values for a 25-g mouse model were obtained from the Olinda code, version 2.2.

### 2.21. Xenograft model: subcutaneous injection, preclinical drug testing, and survival

All animal experiments have been approved by the institutional animal care and use committee of the National Institute of Nuclear Research (ININ). Six-week-old nu/nu male mice were used. HCT116 cells (1 × 10^6^) were resuspended in 100 μL of PBS and injected subcutaneously into the right flanks of nude mice. When tumors reached an average volume of 150 mm^3^, mice were randomly assigned to four groups: Control (untreated), RGF, ^177^Lu-PLGA(RGF) and ^177^Lu-PLGA(RGF)-CXCR4L. Treatments were administered intravenously (i.v), in a final volume of 100 μL. The intravenous administrations were performed on day 1 and day 7. Tumor volume was measured every 3 days and estimated according to the formula V = D × *d*^2^/2, with *d* and D being the shortest and longest diameters, respectively (23). On the first day of treatment, the tumor volumes were normalized to 1 in order to report the relative tumor volume. After 12 days, mice were sacrificed and tumors, lungs, liver, and kidneys were extracted for histological assessment.

For survival evaluation, 12 mice were also inoculated with 1 × 10^6^ HCT116 cells. When tumors reached 150 mm^3^ (~14 days) mice were randomly assigned to four groups, control (without treatment), RGF, ^177^Lu-PLGA(RGF), and ^177^Lu-PLGA(RGF)-CXCR4L. Mice were monitored daily to register mortality for 70 days.

### 2.22. X-ray, radioisotopic, and reflectance preclinical optical images

Images were obtained from mice with the *in vivo* Xtreme imaging system (Bruker, Billerica, MA, United States). For this purpose, mice were anesthetized during radiopharmaceutical administration and image acquisition with a mixture of isoflurane (2%) and oxygen. The X-ray images were acquired using a voltage of 45 kVp, a current of 497 μA and an aluminum filter of 0.8 mm. The exposure time was 1 s, binning was set at 1 × 1 and the field of view (FOV) was 12.5 cm. Radioisotopic images were acquired with an ultrathin, uniform radioisotopic phosphor screen and a parallel-hole collimator. The exposure time was 3 min, with a binning of 4 × 4 and a FOV of 12.5 cm. Reflectance images were acquired using a standard exposure and the high-velocity mode. The exposure time was 1 s, binning 1 × 1 and a FOV of 12.5 cm. Radiation absorbed dose of tumors were estimated after 24 h post-injection of ^177^Lu-PLGA(RGF) or ^177^Lu-PLGA(RGF)-CXCR4L from radioisotopic images.

### 2.23. Immunohistochemistry

Paraffin sections of colon tumor samples were deparaffinized in xylene and rehydrated in a series of graded alcohols. The antigen was retrieved in 0.01 M EDTA-Tris buffer. Samples were incubated in 0.9% H_2_O_2_ for 5 min, followed by 1 h of blocking in 1% BSA in PBS. Next, slides were incubated for 1 h at room temperature with anti-Ki67 (1:100; BIOCARE Medical), washed and set in MACH1 Universal HRP-polymer for 1 h at room temperature. Then, samples were developed with Betazoid DAB Chromogen, counter-stained with hematoxylin and mounted with synthetic resin solution.

### 2.24. Histological analyses of tissue sections by hematoxylin and eosin staining

Mice tumors, liver, kidneys, and lungs were fixed in 4% formaldehyde for 24 h at room temperature, then embedded in paraffin. Tissues were sectioned and stained with hematoxylin and eosin (H&E) using standard protocols. Images were acquired using a Motic AE 2000 microscope (MI01MICRLP), equipped with a Moticam 5.0 MP digital camera.

### 2.25. Statistical analysis

The significance between treatments was analyzed quantitatively via a two-way ANOVA using GraphPad Prism software. Uptake, internalization, cytotoxicity, proliferation, % of apoptotic cells and relative tumor volume results were reported as the mean value and standard error of the mean (SEM). In addition, significance was evaluated by performing the Student’s test (significance *p* < 0.05).

## 3. Results and discussion

In this research, a new nanoparticle system based on Regorafenib (RGF) was designed and synthesized as a chemotherapeutic agent entrapped in PLGA nanoparticles. In addition, ^177^Lu was used as the radiotherapeutic component to produce a synergistic anticancer effect specifically targeted for cancer cells through the CXCR4 ligand.

### 3.1. Preparation of PLGA and PLGA(RGF) nanoparticles

**Figure 2:**
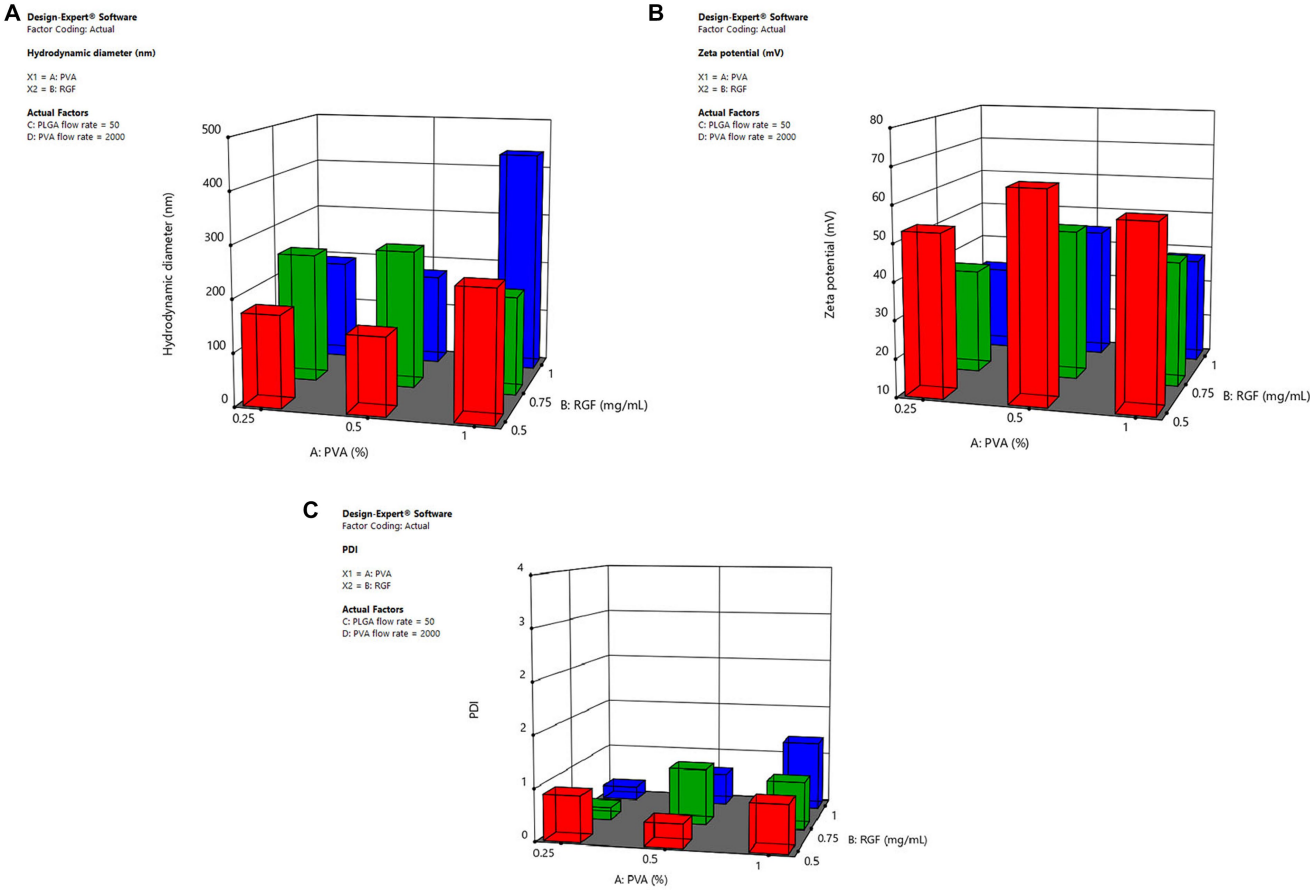
Influence of the selected factors for the optimal nanoparticle formulation. **(A)** Hydrodynamic size, **(B)** ζ potential, and **(C)** Polydispersity index (PDI). PLGA nanoparticles were produced via a microfluidic device, by adjusting PLGA (10% w/v) and PVA (0.25, 0.5, 1, and 1.5% w/v) concentration and controlling the flow rates of the continuous and dispersed phases. The flow rates oscillate between 50/1,000 and 100/1,000 (organic/aqueous phases). The concentration of RGF was 0.5, 1, and 1.5 mg/mL in acetone. Three batches were prepared and the stability was followed by measuring the hydrodynamic diameter and *z* potential.

Microfluidic systems have been developed to synthesize polymeric nanoparticles in a consistent and reproducible way to facilitate and expand the nanoparticle production process. Furthermore, they are economical, reproducible, and modifiable (24). Previous experiments were performed to optimize the nanoparticle synthesis using the Dolomite device. The nanoparticle formulation was optimized employing the Design-Expert 11.1.2.0 software. The ANOVA analyses (*p* < 0.05) of factors (PVA/PLGA flow rates, PVA concentration, and RGF concentration) showed that the hydrodynamic diameter of PLGA nanoparticles was mainly affected by PVA and RGF concentration (Figure 2A). Additionally, the ζ potential was influenced by PVA concentration and PVA flow rate (Figure 2B). Finally, the PDI was defined by the PLGA flow rate in the microfluidic device. PVA at 1% was not appropriate for the desired hydrodynamic size, since this condition favored the large-sized particle formation (200–340 nm; Supplementary Figure S1). Moreover, the formulation with the highest ζ potential was obtained at 0.5 mg/mL of RGF (Figure 2B; Supplementary Figure S2). Finally, the PLGA flow rate of 50 μL/min was determined to favor the lowest PDI values (Figure 2C; Supplementary Figure S3).

The lowest ζ potential was achieved with 50/1,000 and 100/1,000 of PLGA/PVA flow rates (μL/min), respectively. However, the use of 0.5% of PVA and 50/1,000 or 100/1,000 (PLGA/PVA) flow rates (μL/min) favored suitable ζ potential (40–60 mV; Supplementary Figure S2). Therefore, the optimal formulation selected consisted of 0.5% PVA (Factor A), 0.5 mg/mL RGF (Factor B), 50 μL/min flow rate PLGA (Factor C), and 1,000 μL/min flow rate for PVA (Factor D). In good agreement with different PLGA nanoparticle formulations synthesized by using a chip-assisted microfluidic device, it was observed that the lower flow rate of the organic/aqueous phase produced smaller-size nanoparticles (25).

### 3.2. Entrapment efficiency

The RGF loading was performed via microfluidics during the preparation of PLGA nanoparticles. Since the ζ potential was favored by RGF concentration, the optimal preparation condition was set at 0.5 mg/mL, which was also the formulation that exhibited colloidal stability for 2 weeks. The RGF entrapment efficiency of PLGA(RGF) was 91.46 ± 0.47%, (*n* = 3) calculated from the HPLC linear fitting (*y* = 5.2007E^7^–2.038E^6^, *R*^2^ = 0.948). The entrapment efficiency of RGF onto DOTA-PLGA(RGF)-CXCR4L was 91.58 ± 0.22% (*n* = 3). From these results, it was observed that controlling and monitoring the colloidal stability during functionalization is a useful strategy to avoid the irreversible aggregation and changes in the polymeric matrix.

### 3.3. Characterization by DLS and TEM

**Figure 3:**
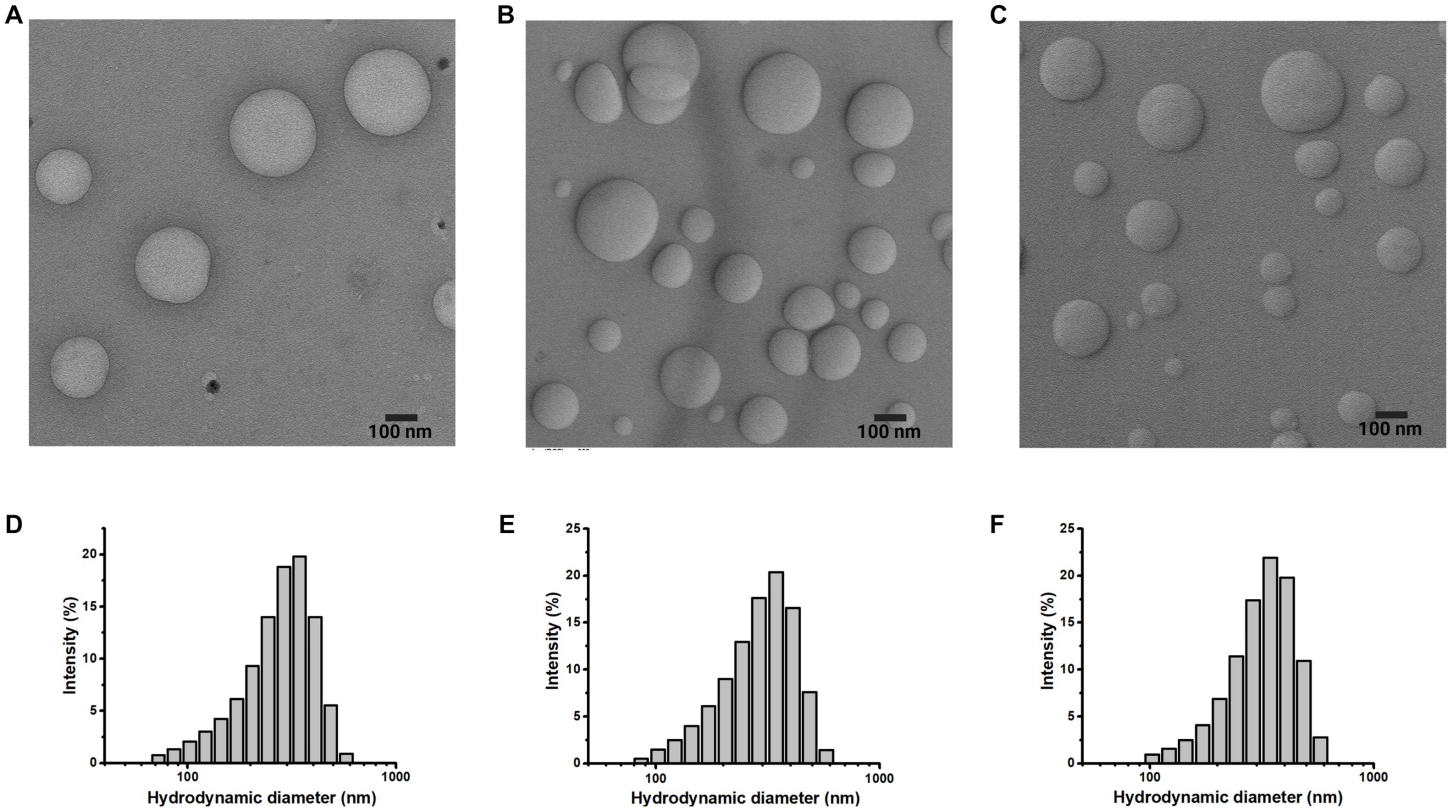
Morphology and size of the nanoparticle system. TEM micrographs and hydrodynamic distribution of **(A)**, **(D)** DOTA-PLGA(RGF), **(B,E)** PLGA(RGF)-CXCR4L, **(C,F)** DOTA-PLGA(RGF)-CXCR4L.

To evaluate the effect of adding molecules to the polymeric systems, the morphology, size, and distribution were analyzed via TEM and DLS, respectively. The TEM micrographs showed adequate dispersion of nanoparticles with spherical shape. Furthermore, during the surface modification of nanoparticles (Figure 1), there were no morphological differences (Figures 3A–C).

The mean hydrodynamic diameter of PLGA and PLGA(RGF) nanoparticles was 129.7 ± 50.2 and 141.9 ± 77.0 nm, respectively (Table 1), with a wide distribution of an apparent monomodal population [PDI = 0.263 for PLGA and 0.559 for PLGA(RGF)]. RGF loading allowed an increase in hydrodynamic diameter (in an aqueous solution), which was attributed to the rearrangements in the inner polymer due to the RGF encapsulation.

**Table 1:** The hydrodynamic diameter of nanoparticle systems.

Nanoparticle system	Size (nm)	Polydispersity Index (PDI)	Z potential (mV)
PLGA	129.7 ± 50.2	0.263	31.0
PLGA(RGF)	141.9 ± 77.0	0.559	52.9
DOTA-PLGA	197.6 ± 61.0	0.161	41.9
DOTA-PLGA(RGF)	265.3 ± 97.2	0.514	61.2
PLGA-CXCR4L	276.6 ± 94.9	0.367	57.2
PLGA(RGF)-CXCR4L	278.6 ± 99.4	0.402	58.0
DOTA-PLGA-CXCR4L	277.0 ± 97.9	0.205	75.3
DOTA-PLGA(RGF)-CXCR4L	280.0 ± 97.2	0.347	80.8

The chelating agent DOTA and the peptide sequence CXCR4L were conjugated to PLGA or PLGA(RGF) nanoparticle surface via the carboxylate activating agent HATU. Hydrodynamic diameter of DOTA-PLGA(RGF) was 265.3 ± 97.2 nm (Figure 3D), and the diameters of PLGA(RGF)-CXCR4L and DOTA-PLGA(RGF)-CXCR4L systems were 278.6 ± 99.4 nm (Figure 3E) and 280.0 ± 97.2 nm (Figure 3F), respectively. It has been shown that particles between 100 and 300 nm are prepared successfully to transport drugs, via the exploitation of the enhanced permeability and retention effect in cancer cells (26).

The hydrodynamic diameter measurement of conjugated systems was higher than that obtained for PLGA and PLGA(RGF). The increase in hydrodynamic diameter was due to the presence of DOTA, CXCR4L, or both on the surface of the PLGA nanoparticle.

### 3.4. Characterization by FT-IR

**Figure 4:**
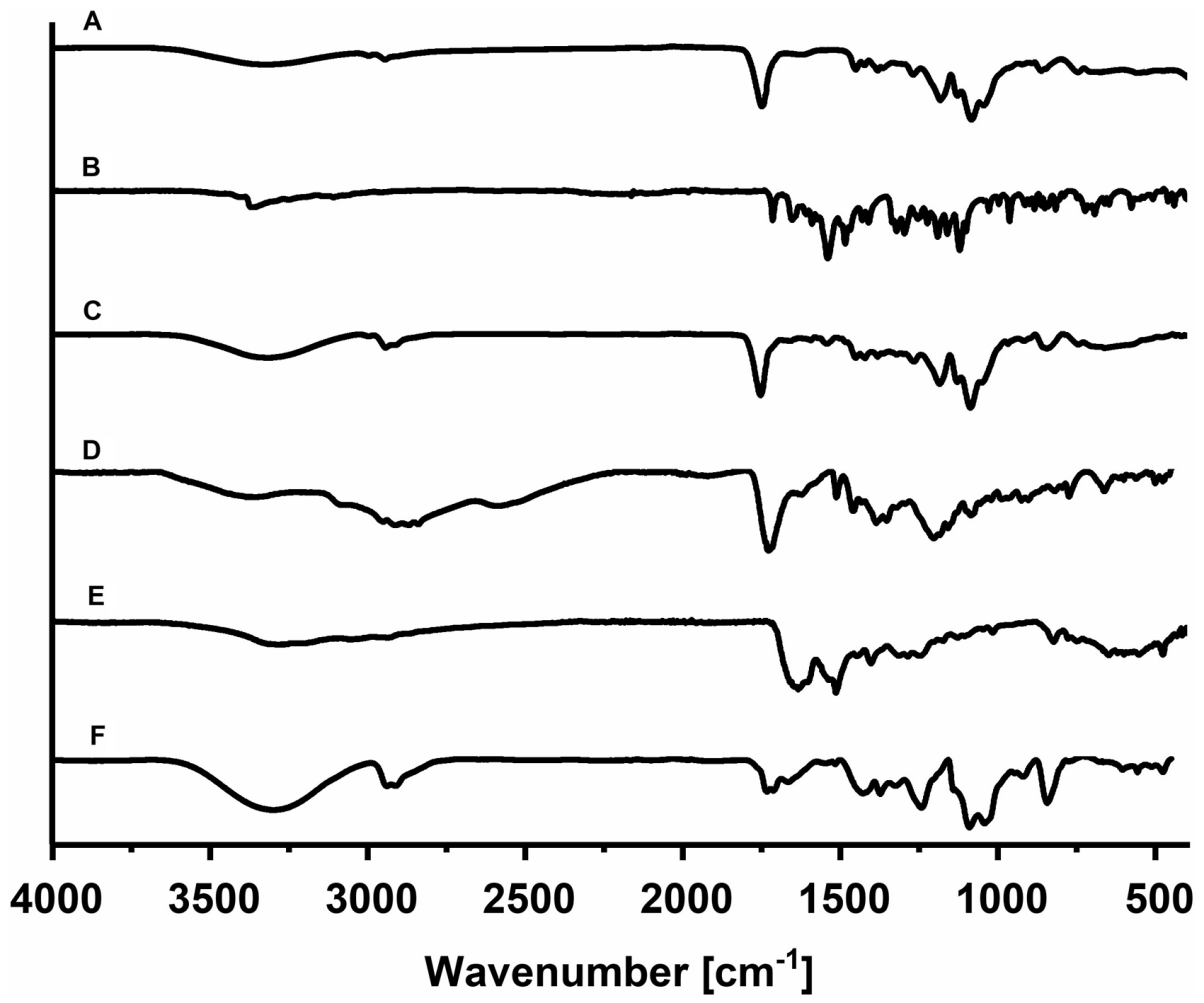
The comparative infrared spectrum of individual components: **(A)** PLGA NPs, **(B)** RGF, **(C)** PLGA(RGF), **(D)** p-NH_2_-Bn-DOTA, **(E)** HYNIC-CXCR4L, and **(F)** DOTA-PLGA(RGF)-CXCR4L.

The PLGA nanoparticles spectrum showed vibrational modes corresponding to copolymer characteristic groups: 2,945 cm^−1^ (CH bend), 1,749 cm^−1^ (C=O of ester), 1,383 cm^−1^ (-CH_3_ from lactide), and 1,300–1,000 cm^−1^ (ester). A triple-peak absorption pattern is also present, corresponding to bonds between monomeric units of lactide-lactide (L-L) at 1,455 cm^−1^, glycolide-glycolide (G-G) at 1,425 cm^−1^, and lactide-glycolide (L-G) at 1,383 cm^−1^, within the PLGA polymer chains (Figure 4A). Also, characteristic PVA bands were seen at 3,324 cm^−1^, belonging to the -OH group from the alcohol (27). In the FT-IR spectra of RGF, an absorption band at 3,358 cm^−1^ corresponds to N-H stretching, 3,111 cm^−1^ corresponds to C-H stretching, and 1,715 cm^−1^ corresponds to C=O stretching. The bands at 1,658, 1,544, and 1,486 cm^−1^ correspond to aromatic ring stretch bands (Figure 4B) (28).

The FT-IR spectrum corresponding to Regorafenib-loaded nanoparticles showed no difference concerning the empty nanoparticle spectrum. These spectra did not display the characteristic intense bands from free RGF; the bands produced by the polymer may have masked them. The possible absence of chemical interaction between the polymer and drug may indicate a complete encapsulation of RGF into the nanoparticles (Figure 4C). On the other hand, the p-NH_2_-Bn-DOTA FT-IR-spectrum showed a well-defined band at 1,921 cm^−1^, which was assigned to the isothiocyanate motif vibration. At 2,952–2,838 and 1,400 cm^−1^ region, vibrations attributed to the C-H stretching appear, and at 3,346 cm^−1^, the presence of amine groups was identified. At 1,729 cm^-1^, C=O stretching is shown and at 1,513 cm^−1^, the aromatic C-C stretching was also observed (Figure 4D). The spectrum of CXCR4L showed a wide band, which was centered at 3,200 cm^−1^, attributed to N–H vibration from the free amines of the peptide sequence (Tyr, Orn, Arg, and Gly) and the C–H vibrational mode at 3,045 cm^-1+^, from the aromatic rings (tyrosine, naphthyl, and HYNIC motif), was found. At 1,635 and 1,514 cm^−1^, there were characteristic vibrations from the C–C bond of the aromatic rings (tyrosine, a naphthyl group, and HYNIC). Between 822 and 660 cm^−1^, a highly-structured spectrum can be observed because of the substitutions in the aromatic rings. Finally, at 1,190 and 1,126 cm^−1^, two high-intensity bands were present due to the C–N bond vibrations of the tertiary amines found in the DOTA macrocycle (Figure 4E) (14).

The infrared spectrum corresponding to the final system DOTA-PLGA(RGF)-CXCR4L, showed each component’s contribution of characteristic vibrations. The increased and centered region at 3,300 cm^−1^ is assigned to the amines present in the macrocycle, while the ester region displayed and increased confirmed the presence of CXCR4L and DOTA (Figure 4F).

### 3.5. Characterization by UV-Vis

**Figure 5:**
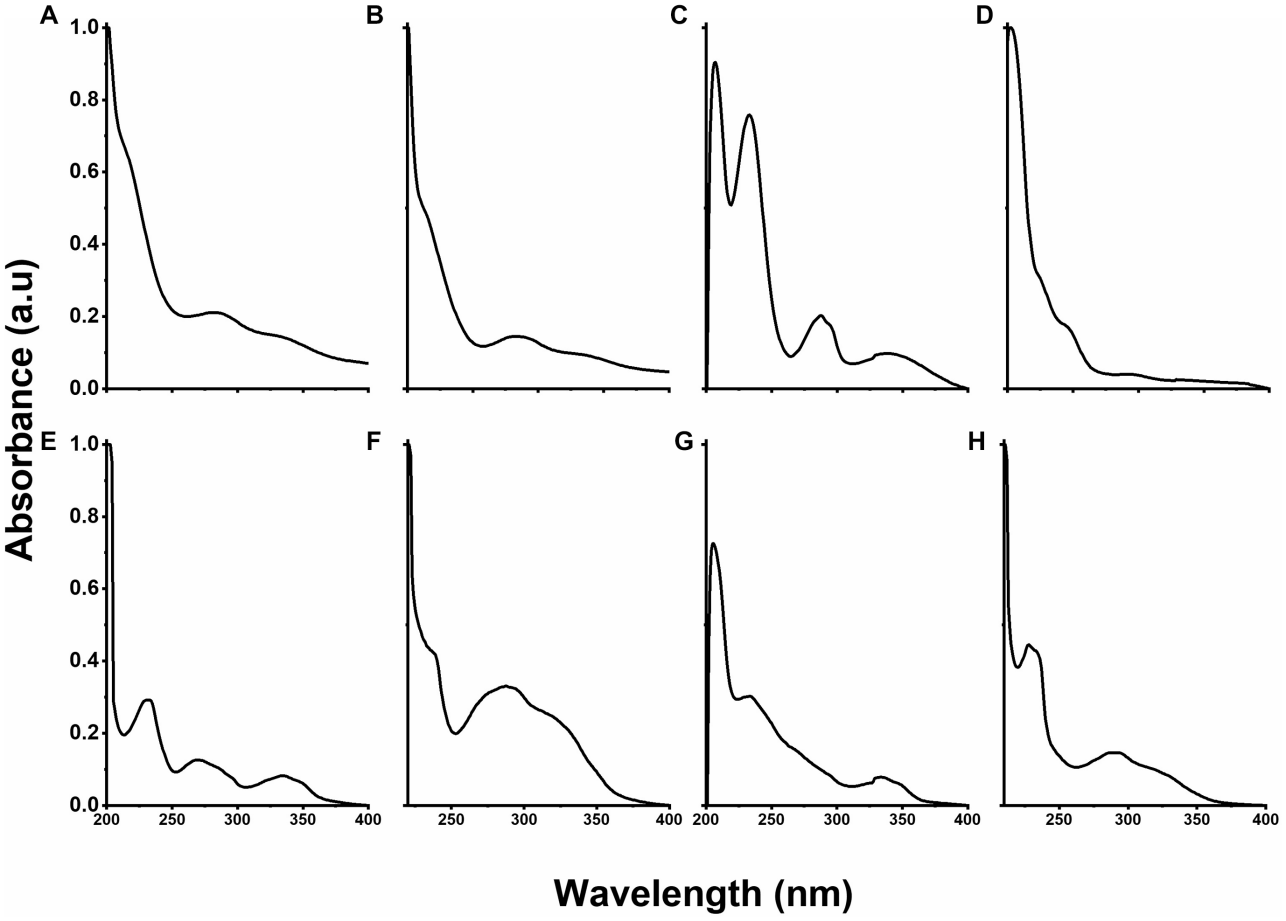
UV–Vis optical density normalized spectra of: **(A)** PLGA, **(B)** PLGA(RGF), **(C)** DOTA-PLGA, **(D)** DOTA, **(E)** PLGA-CXCR4L, **(F)** PLGA(RGF)-CXCR4L, **(G)** DOTA-PLGA(RGF), and **(H)** DOTA-PLGA(RGF)-CXCR4L.

The UV-Vis spectrum of PLGA nanoparticles showed absorption bands centered at 212, 285, and 333 nm (Figure 5A), whereas the contribution of RGF to the formation of PLGA(RGF) was observed in Figure 5B, at the absorption band centered at 285 nm, a band that was overlapped with those from PLGA nanoparticles (21, 27). The UV-Vis spectrum of the DOTA-PLGA showed absorption bands at 207, 232, 287, and 341 nm (Figure 5C). The first one corresponds to the –COOH group from the DOTA molecule (Figure 5D) and the others represented the contribution of PLGA nanoparticles (Figure 5A) (29). The PLGA-CXCR4L spectrum showed absorption bands at 230, 270, and 335 nm (Figure 5E). When the RGF-loaded PLGA nanoparticles were modified with the moieties DOTA and CXCR4L, the spectra of PLGA(RGF)-CXCR4L and DOTA-PLGA(RGF) (Figures 5F,G, respectively) showed a bathochromic shift in the absorption bands centered at ~230 nm (from 232 to 233 for DOTA-PLGA nanoparticles and from 230 to 236 for PLGA-CXCR4L nanoparticles) and hypochromic bands as a result of overlapping $n\to \pi^{*}$ electronic transitions originated from the carbonyl groups of PLGA and RGF. The rest of the absorption bands in those systems had a hypochromic displacement, indicating a change in the chemical composition and the molecules surrounding media. Figure 5H shows the spectrum of the final system, DOTA-PLGA(RGF)-CXCR4L nanoparticles, with absorption bands centered at 227, 234, 291, and 322 nm, which correspond to the interactions from the different components due to their $n\to \pi^{*}$ and $\pi \to \pi^{*}$ transitions, where the characteristic band of CXCR4L was still centered at 322 nm. Moreover, the broad bands in the spectrum also indicated the contribution of the unsaturation of each component to form the final structure, suggesting the correct formation of the DOTA-PLGA(RGF)-CXCR4L nanosystem.

### 3.6. Nanoparticles radiolabeling

**Figure 6:**
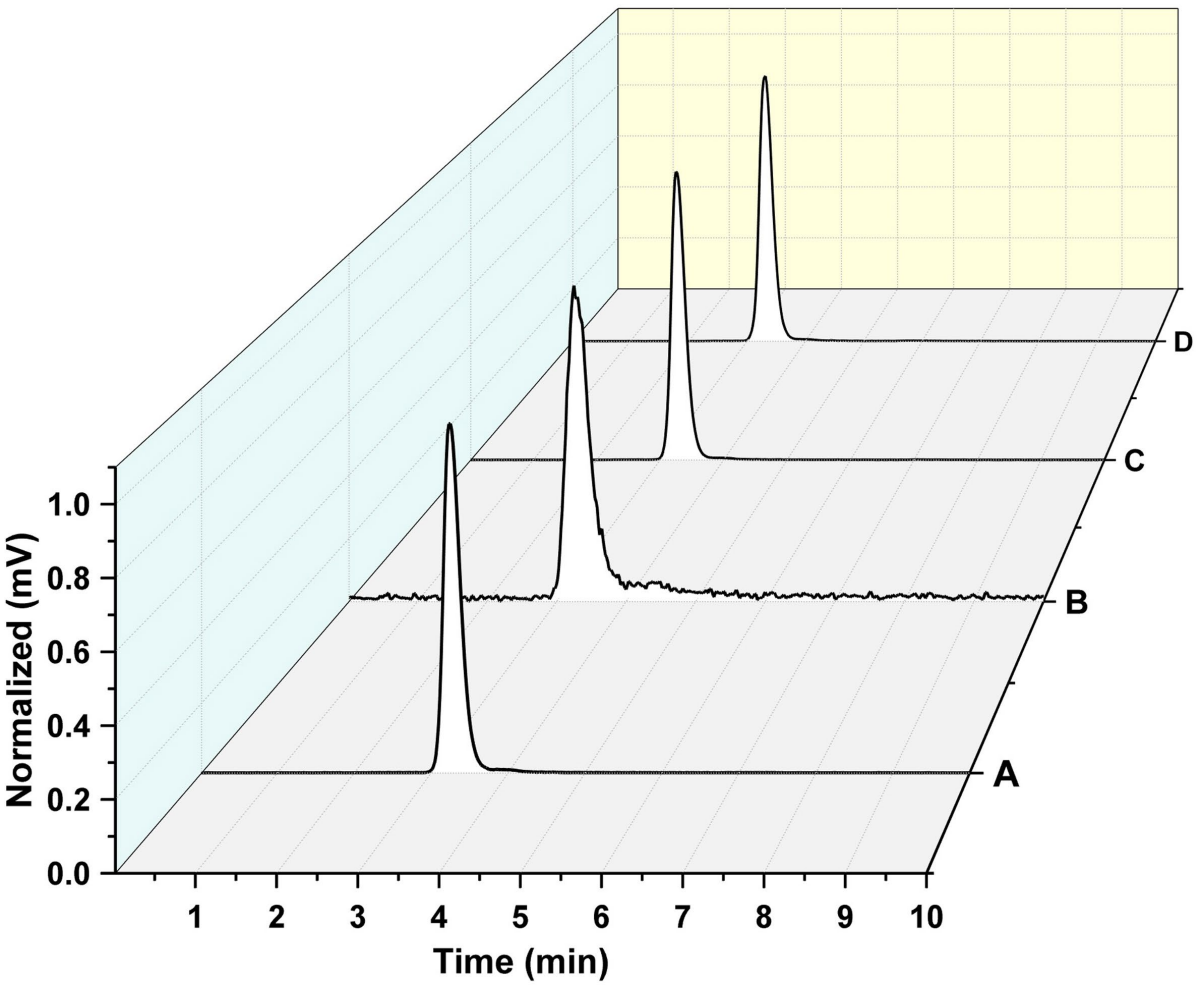
Radiochemical purity. Size-exclusion radio-HPLC chromatogram of **(A)** ^177^Lu-PLGA, **(B)** ^177^Lu-PLGA-CXCR4L, **(C)** ^177^Lu-PLGA(RGF), and **(D)** ^177^Lu-PLGA(RGF)-CXCR4L.

The radiolabeling efficiency was evaluated through radiochemical purity for all the intermediates and the final system. The ^177^Lu-PLGA (Figure 6A), ^177^Lu-PLGA-CXCR4L (Figure 6B), ^177^Lu-PLGA(RGF) (Figure 6C), and ^177^Lu-PLGA(RGF)-CXCR4L (Figure 6D) radiochemical purity was >99% as evaluated by size-exclusion HPLC (MeOH:ACN:H_2_O 60:20:20 v/v) with a flow rate of 2 mL/min. The retention time was 3.1–3.3 min. Under these conditions, ^177^Lu remains in the column, as corroborated by ITLC-SG, using the same mobile phase (Rf = 0 for ^177^LuCl_3_ and Rf = 0.8 for ^177^Lu-PLGA^, 177^Lu-PLGA(RGF) and ^177^Lu-PLGA-CXCR4L). No differences in retention time were observed among the intermediates, which is attributed to the relatively small changes in molecular weight of the final system resulting from the binding of CXCR4L (837 g/mol) or DOTA (568 g/mol) molecules on the total surface of the nanoparticle (approximately 250 kDa, as measured by SDS-PAGE).

The radiochemical purity of ^177^Lu-PLGA(RGF)-CXCR4L was also corroborated by ITLC-SG using NaCl (0.9%): 0.2 M HCl (Rf = 1.0 for ^177^LuCl_3_), where the Rf was 0. Meanwhile, using MeOH:ACN:H_2_O 60:20:20 v/v as mobile phase, the Rf for ^177^Lu-PLGA(RGF)-CXCR4L was 0.8.

Five hours post-radiolabeling, ^177^Lu-PLGA(RGF)-CXCR4L remained unchanged, which demonstrated that this radiopharmaceutical could be prepared from a lyophilized formulation and be kept in solution 5 h after preparation.

### 3.7. Nanoparticle stability

**Figure 7:**
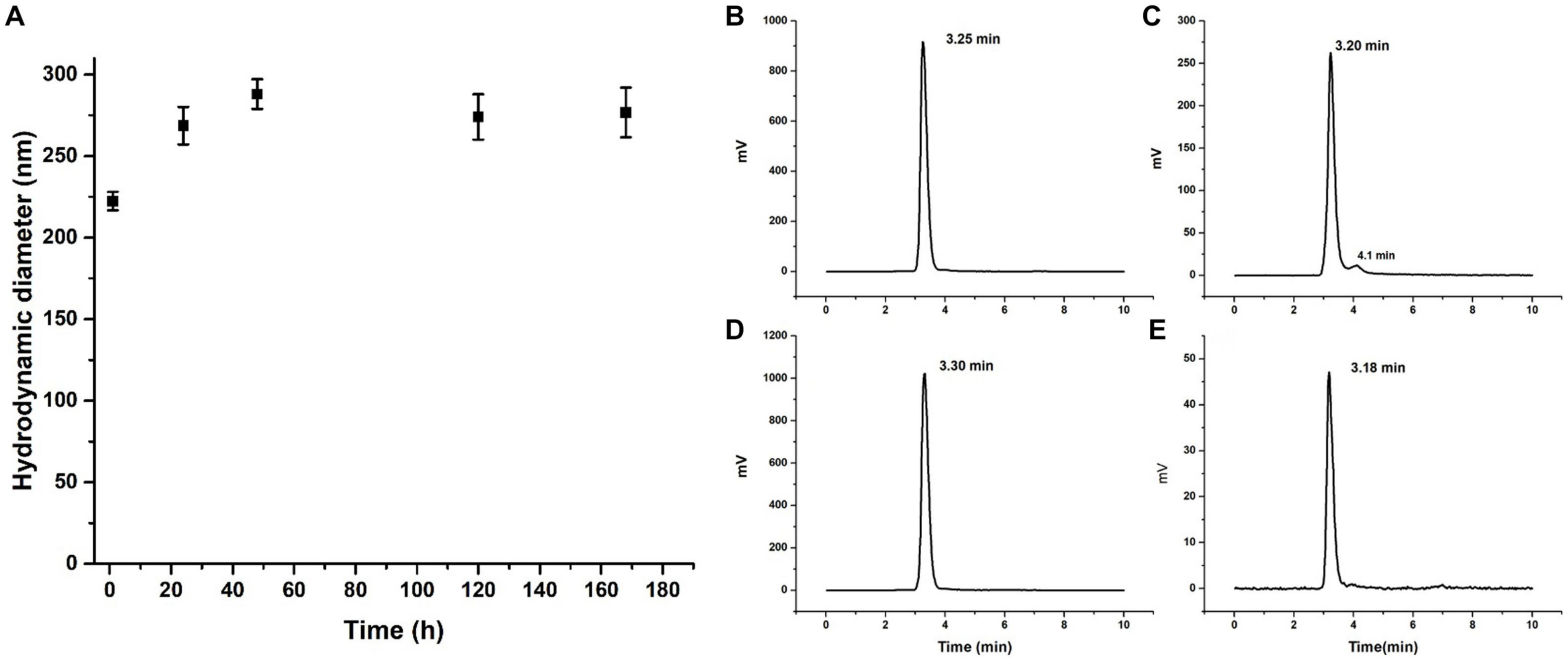
Nanoparticle stability. **(A)** Hydrodynamic diameter of DOTA-PLGA(RGF)-CXCR4L at 1, 24, 48, 120, and 168 h after reconstitution. Radiochromatogram of ^177^Lu-PLGA(RGF) after radiolabeling **(B)**, 5 h after radiolabeling **(C)**. Radiochromatogram of ^177^Lu-PLGA(RGF)-CXCR4L after radiolabeling **(D)**, 5 h after radiolabeling **(E)**.

Lyophilized DOTA-PLGA(RGF)-CXCR4L nanoparticles (1 mg) were fully redispersed 1 year after lyophilized in 0.5% PVA solution at established conditions (1 mL PVA, 37°C, 3 h in thermo-shaker). This formulation rendered sizes of 222.3 ± 5.6 nm (*n* = 3). No additional cryoprotectant other than PVA was required (Figure 7A). Seven days later, fully redispersed, the hydrodynamic diameter of the same solution was measured again and sizes of 267 ± 30.1 nm (*n* = 3) were obtained (Figure 7A).

When radiolabeled, DOTA-PLGA(RGF) and DOTA-PLGA(RGF)-CXCR4L, from their lyophilized formulations, produced 97–100% radiochemical purity without purification, as measured by ITLC-SG (Supplementary Figures S1A–D) and HPLC (Figures 7B,C). It was observed that DOTA-PLGA(RGF)-CXCR4L remained stable for 5 h after radiolabeling at room temperature (Figures 7D,E). Meanwhile, ^177^Lu-PLGA(RGF) in solution produced a slight separation of greater nanoparticles due to the formation of aggregates (see the additional peak at 4.1 min; Figure 7C). The presented results allow us to assume that the nanoradiopharmaceutical formulation remains stable at 4–8°C in its sealed container system (Type B glass container with elastomeric stopper −20 mm-and aluminum seal) for at least 1 year for further reconstitution.

### 3.8. Uptake and internalization

**Figure 8:**
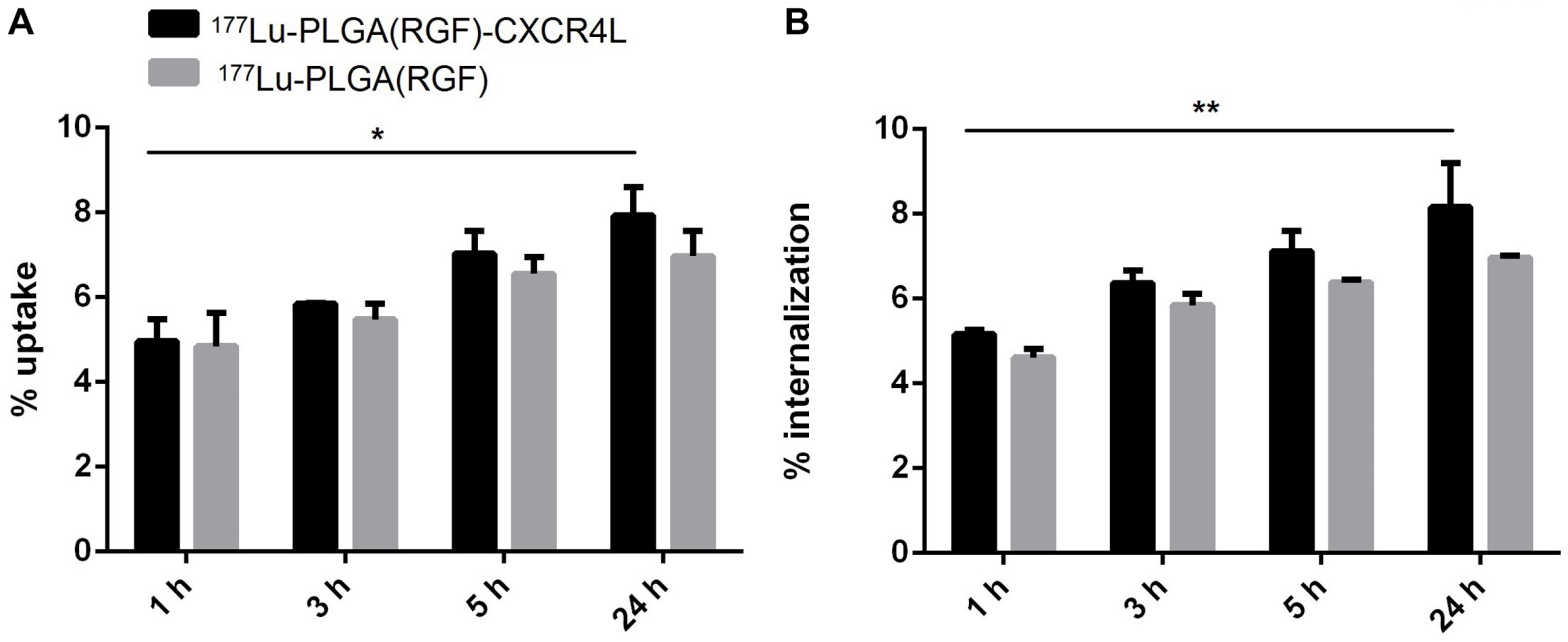
Comparison of **(A)** cell uptake and **(B)** internalization of ^177^Lu-PLGA(RGF) and ^177^Lu-PLGA(RGF)-CXCR4L nanoparticle systems after incubation of 1, 3, 5, and 24 h (^*^p < 0.01, ^**^p < 0.001) in HCT116 colorectal cancer cells.

Nanoparticle systems based on biodegradable polymers such as PLGA are often associated with insufficient cellular uptake by cancer cells due to the lack of a recognition molecule on their surface (30). Moreover, overexpression of different chemokine receptors has been observed in tumor cells, particularly CXCR4, while their neutralization significantly reduces metastasis formation (31). In clinical trials, overexpression of the CXCR4 receptor was associated with a worse prognosis in patients undergoing surgery for colorectal liver metastases (32). We analyzed *in vitro* uptake and internalization of PLGA nanoparticle systems with and without CXCR4L in the HCT116 colorectal cancer cell line at 1, 3, 5, and 24 h. Internalization was calculated from the uptake percentage. Both nanosystems display a time-dependent accumulation in the cellular plasma membrane (Figure 8A) and cytoplasm (Figure 8B). However, no significant differences were observed between ^177^Lu-PLGA(RGF)-CXCR4L and ^177^Lu-PLGA(RGF). This behavior can be explained due to the unspecific uptake attributed to the similar molecular weight, size, and morphology of nanosystems (33) as can be corroborated by size exclusion chromatograms (Figure 6) where differences among the retention times of intermediates products were not observed.

Since, the uptake and internalization of nanoparticles sizing 100 nm are taken up into the cells more efficiently than those with dimensions of 50, 200, 500, and 1,000 nm (34), CXCR4L-targeted nanosystems could be uptaken through receptor-mediated endocytosis and untargeted nanoparticles through the caveolin-mediated endocytic pathway (35).

### 3.9. Survival, proliferation, and cell signaling

**Figure 9:**
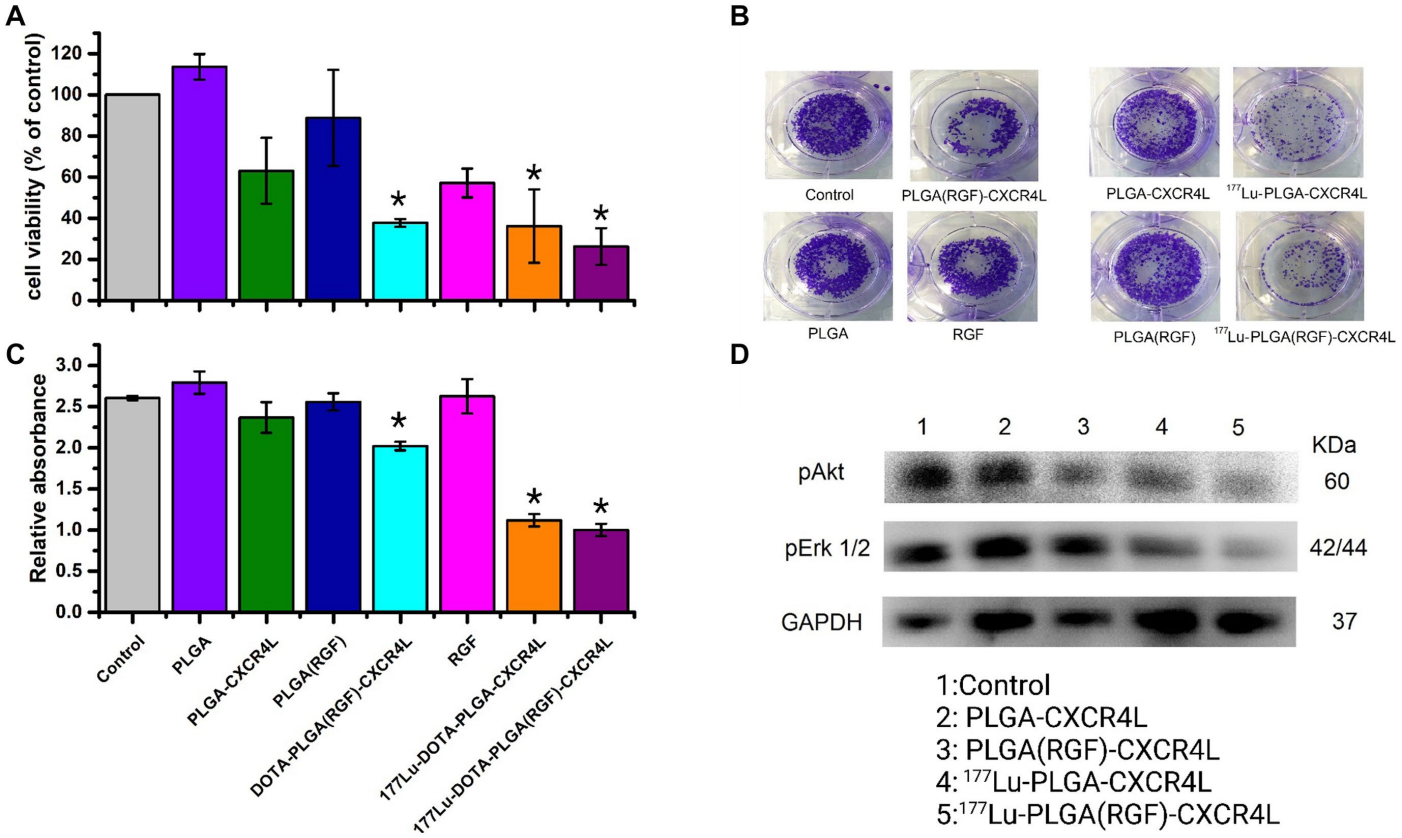
Combined chemo-radiotherapy with the ^177^Lu-PLGA(RGF)-CXCR4L nanosystem inhibits cell survival and proliferation of HCT116 cancer cells through a decrease of Akt and Erk activation. **(A)** The colorectal cancer cell line HCT116 was treated with ^177^Lu-PLGA(RGF)-CXCR4L and its respective controls for 24 h (RGF at a concentration of 7.26 μM and 20 μCi of ^177^Lu). **(B)** Cells were treated with ^177^Lu-PLGA(RGF)-CXCR4L and its respective controls in a clonogenic assay; blue-violet colonies are viable cells. Representative images of the clonogenic assay of three independent experiments are shown. Quantification of crystal violet staining from HCT116 cancer cells depicted in **(C)** *n* = 3. ^***^p < 0.0001. **(D)** WB-analyzed cell lysates for phosphorylation levels of pErk1/2, pAkt, and GAPDH as a loading control. Representative blots of three independent assays are shown.

The cytotoxicity of the nanosystems was evaluated *in vitro* using HCT116 cancer cells. After 24 h of treatment, only the final nanoparticle system [^177^Lu-PLGA(RGF)-CXCR4L] inhibits 74% of cell viability (Figure 9A) and 61% of cell proliferation (Figures 9B,C). Furthermore, the targeting molecule CXCR4L also increases the cytotoxicity of the system because both the ^177^Lu-PLGA-CXCR4L and PLGA(RGF)-CXCR4L systems decrease cell viability to 64.0 and 62.4%, respectively, and proliferation to 57.6 and 38.4%, correspondingly (Figure 9C). Notably, empty PLGA nanoparticles did not show activity in the viability and proliferation of cancer cells, because of their low toxicity and high biocompatibility (7). RGF, at a concentration of 7.26 μM, resulted in a decrease of 42.9% in cell viability (Figure 9C). Moreover, RGF suppresses the activation of extracellular signal-regulated kinase (Erk) and Protein Kinase B (PKB, or Akt) (36, 37). pErk and pAkt are involved in Ras/Raf/MEK/ERK and PI3K/Akt/mTOR pathways, respectively. Consequently, stimulation of these molecular pathways improves cancer cell proliferation, survival or anti-apoptosis, and aberrant metabolism (38). Given the known function of RGF, we next determined whether the nanosystem decreases cell viability and proliferation through inhibition of Erk and Akt activation (pErk1/2 and pAkt, respectively). Furthermore, radiation resistance is enhanced through PI3K/Akt and MEK/Erk signaling and this effect is neutralized by the kinase inhibitors (39). Figure 9D confirms that nanosystems PLGA(RGF)-CXCR4L and ^177^Lu-PLGA(RGF)-CXCR4L significantly reduced the activation (phosphorylation of Ser-473) of pAkt. This figure shows also the decreased activation of Akt and Erk after treatment with ^177^Lu-PLGA-CXCR4L. This response is explained by the effect of the radiation dose delivered from lutetium-177, which produces changes in the expression of some proteins such as SNAIL which affect the phosphorylation of the Erk and Akt signaling pathways (40). Notably, the combined effect of chemo-radiotherapy and the ^177^Lu-PLGA(RGF)-CXCR4L system significantly decreased the activation (Thr202/Tyr204 phosphorylation) of pErk1/2.

### 3.10. Apoptosis

**Figure 10:**
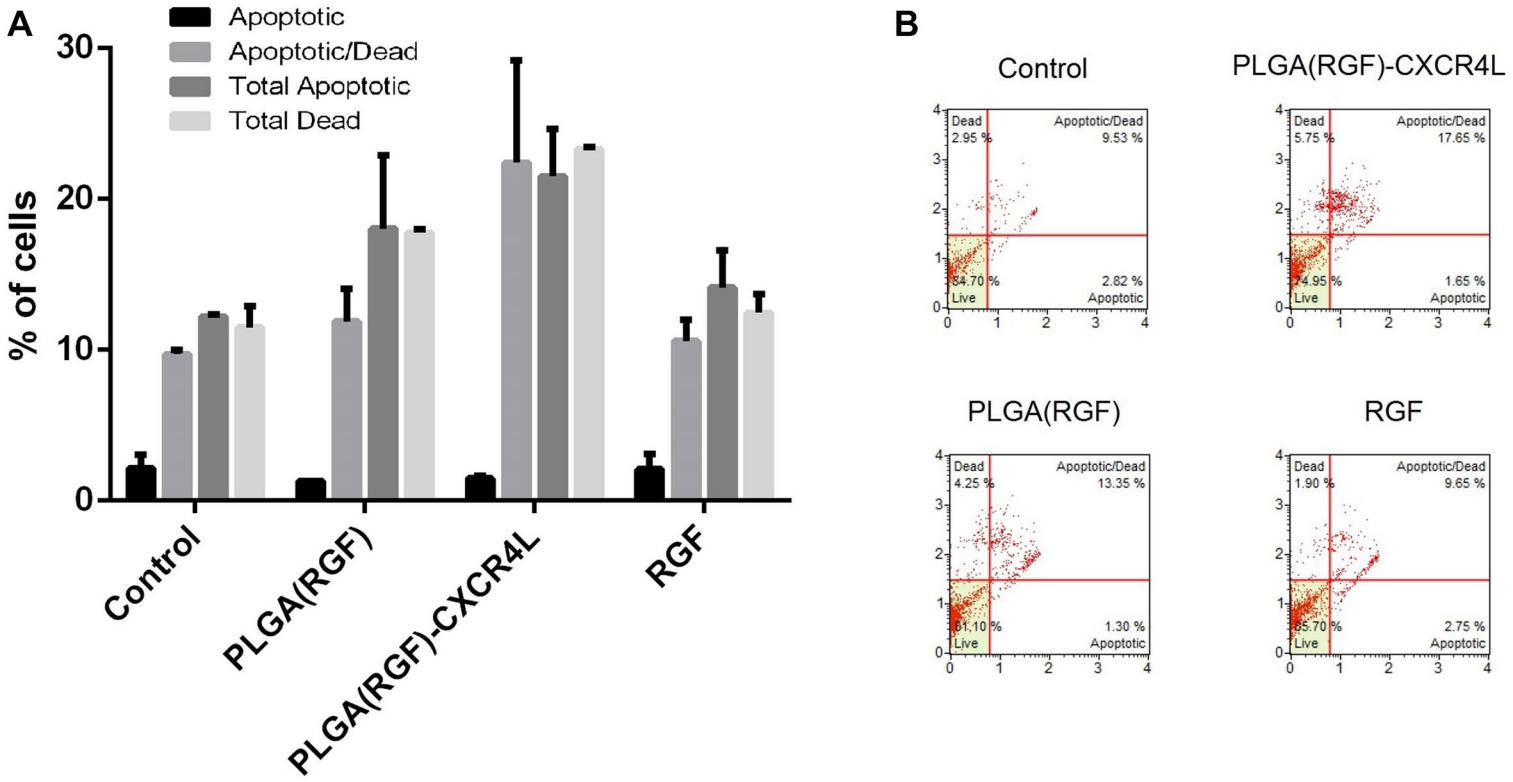
PLGA(RGF)-CXCR4L increase the percentage of apoptotic/dead cells 24 h after treatment. **(A)** Percentage of HCT116 colorectal cancer cells in apoptotic [Caspase-3/7 (+) and 7-AAD (−)] and Late Apoptotic/Dead [Caspase −3/7 (+) and 7-AAD (+)]. Error bars represent SEM. **(B)** Caspase −3/7 and 7-AAD analysis of HCT116 cells treated with PLGA(RGF)-CXCR4L for 24 h.

To analyze the status of apoptosis and cell death of HCT116 cancer cells after 24 h of ^177^Lu-PLGA(RGF)-CXCR4L exposure, the Muse Caspase 3/7 kit (Catalog No. MCH100108) was used. A previous report showed that RGF induces apoptosis through the induction of expression of the p53-upregulated modulator of apoptosis (PUMA) in HCT116 colorectal cancer cells (41). This work assessed apoptosis through activated Caspase 3/7 and cell death was measured by cellular plasma membrane permeabilization with 7-Aminoactinomycin (7-AAD). Relative percentages of apoptotic cells exhibiting: Caspase-3/7 activity (+) and 7-AAD (−), and Late Apoptotic/Dead cells: Caspase −3/7 (+) and 7-AAD (+) (Figure 10A). Treatment with PLGA(RGF)-CXCR4L leads to an increase in 17.65% of cells in the stage of late apoptotic/death, which corroborates the decrease in viability and proliferation of HCT116 cells. In contrast, the PLGA(RGF) treatment results in an increase of 13.35% of cells in late apoptotic/death. In comparison, free RGF exhibits an increase of 9.65% of cells in late apoptotic/dead (Figure 10B). On the other hand, PLGA nanoparticles alone did not significantly affect cell dead (8.25%), when compared to the control cells (9.53%; Figures 10A,B).

### 3.11. Biodistribution and dosimetry

**Figure 11:**
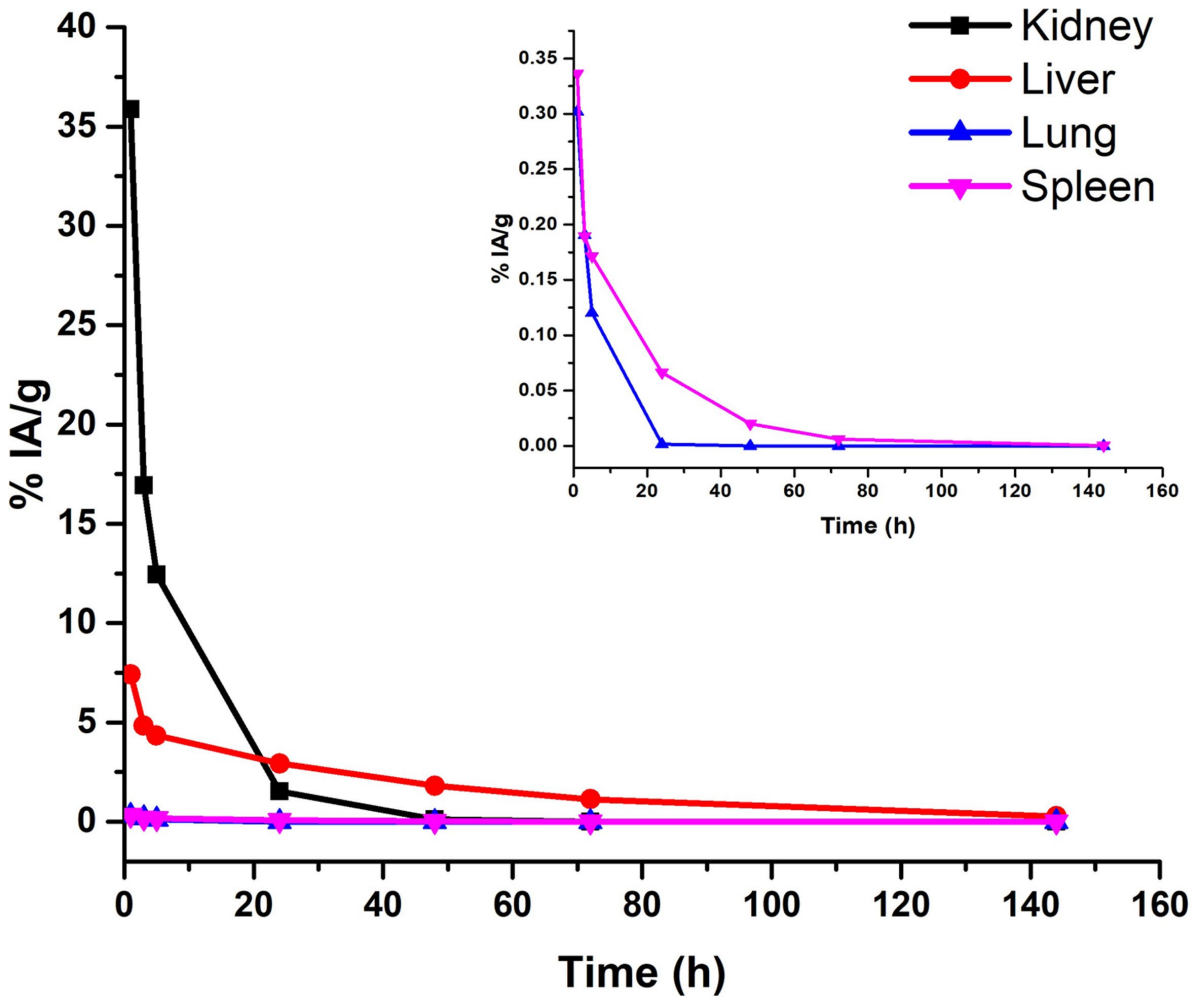
Biokinetic profile of ^177^Lu-PLGA(RGF)-CXCR4L [A_h_ (t)] in BALB/c mice after intravenous administration.

The biodistribution profile of ^177^Lu-PLGA(RGF)-CXCR4L was evaluated at 3, 5, 24, 48, and 144 h. After iv administration, renal and hepatobiliary excretion were observed (Figure 11). The improved renal excretion can be explained based on the fact that PLGA polymers are degraded by esterase enzyme forming lactic and glycolic acids, which are then incorporated into the Krebs cycle and eliminated as CO_2_ and water through respiration, feces, and urine (Figure 11, inset) (42, 43).

As can be calculated from the biokinetic models, in Table 2, 91% of ^177^Lu-PLGA(RGF)-CXCR4L was cleared from kidneys within the first hour after administration (λ = 1.17 h^−1^) and 9% of nanoradiopharmaceutical was removed within 6 h. In the tumor, this behavior clearly contrasts with the first eliminated fraction of 93% at 5.3 h (λ = 0.13 h^−1^). The second component (7%) was cleared from the tumor after 69 h (Table 2). These results also correlated with the radiation absorbed dose in the tumor (1.12 Gy/MBq) if compared with 0.37 Gy/MBq in the kidney or 0.09 Gy/MBq produced in the liver.

**Table 2:** Total nuclear transformations in source organs (N).

Source organs	Models A(t)	N (MBq·h/MBq)	^177^Lu-PLGA(RGF)-CXCR4L (Gy)
Kidney	$A\left(t\right)=-54.1e^{-1.17t}+21.3e^{-0.11t}\;$ R^2^ = 1	1.37	0.37
Liver	$A\left(t\right)=7.71e^{-1.02t}+4.74e^{-0.02t}\;$ R^2^ = 1	1.85	0.09
Lung	$A\left(t\right)=0.79e^{-9.61t}+0.38e^{-0.23t}\;$ R^2^ = 1	0.01	0.02
Spleen	$A\left(t\right)=-4.61e^{-3.59t}+0.22e^{-0.05t}\;$ R^2^ = 0.99	0.03	0.02
Tumor	$A\left(t\right)=1.72e^{-0.13t}+2.16e^{-0.01t}\;$ R^2^ = 1	1.79	1.12

Organ-absorbed doses (Gy per 1 MBq administered) in mice after ^177^Lu-PLGA(RGF)-CXCR4L administration (S-values for 25 g mouse phantom, OLINDA 2.1).

Previous reports have demonstrated that *in vivo* behavior of nanoparticles is initially dictated by the enhanced permeability and retention effect, which takes advantage of the endothelial intercellular formed gaps ranging from 400 to 800 nm in the tumor. Therefore, ^177^Lu-PLGA(RGF)-CXCR4L and ^177^Lu-PLGA(RGF), with round nanometric sizes (280 ± 97.2 and 278 ± 99 nm, respectively), will primarily accumulate in the tumor stroma (44–46).

Once inside the tumor microenvironment, ^177^Lu-PLGA(RGF)-CXCR4L is additionally uptake and internalized by specific mechanisms mediated by the CXCR4 protein, which is also overexpressed in cancer cells. This result was corroborated by the tumor-absorbed dose, which was higher for ^177^Lu-PLGA(RGF)-CXCR4L nanosystem (1.2 Gy), when compared with the ^177^Lu-PLGA(RGF) nanosystem (0.4 Gy) according to the biokinetic model ( $A\left(t\right)=-1.72e^{-0.13t}+2.16e^{-0.01t}$ , $\;A\left(t\right)=-3.10e^{-0.09t}+0.46e^{-0.02t}$ , respectively; Table 2).

The targeted nanosystem showed increased uptake in the kidney and liver at 1 h. In the lung and spleen, the average percentage of injected radioactivity (%IA) of ^177^Lu-PLGA(RGF)-CXCR4L decreased in parallel. The kidney was the organ that received the highest radiation dose per MBq of radioactivity administered (0.37 Gy); the liver received 0.09 Gy; the lungs and spleen received 0.02 Gy (Table 2).

Since CXCR4 is also overexpressed in hematological, breast, esophageal, head and neck, renal, lung, gynecologic, liver, prostate, and gallbladder cancers (47), ^177^Lu-PLGA(RGF)-CXCR4L would also be a potential nanoapproach to treat these malignancies at the clinical level.

### 3.12. Preclinical assessment

**Figure 12:**
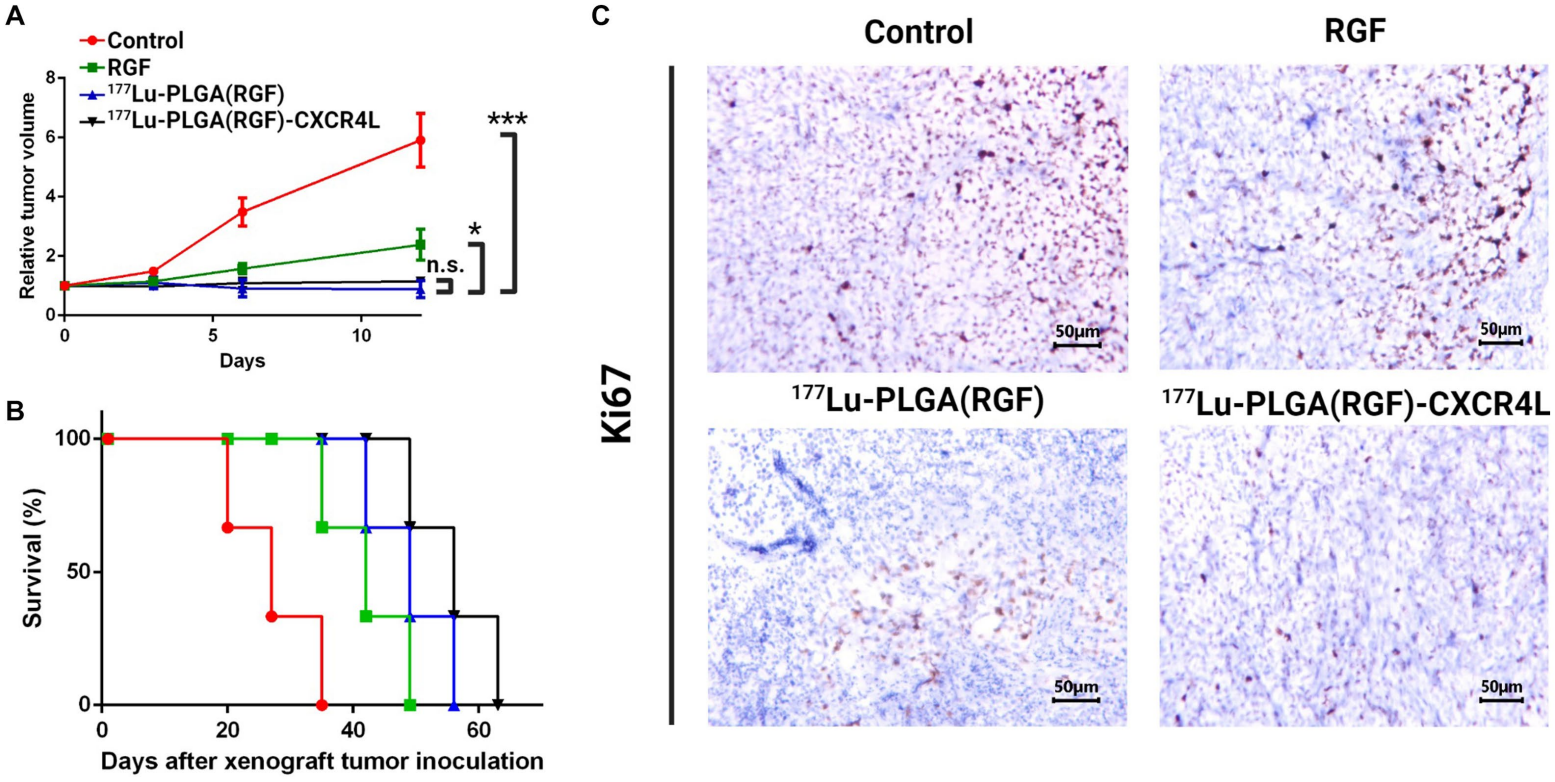
Therapeutic *in vivo* efficacy of the ^177^Lu-PLGA(RGF)-CXCR4L nanosystem. **(A)** Twice -intravenously-administered treatments, with and without CXCR4L, reduce the tumor size in murine colorectal cancer xenografts, compared to free RGF and control (untreated). *n* = 3; ^***^p < 0.001. Error bars represent SEM. **(B)** Survival rate for each experimental group. **(C)** Representative images of immunohistochemical staining for Ki67 (a marker of proliferation) in the xenograft tumor tissues. The scale bar represents 200 μM.

**Figure 13:**
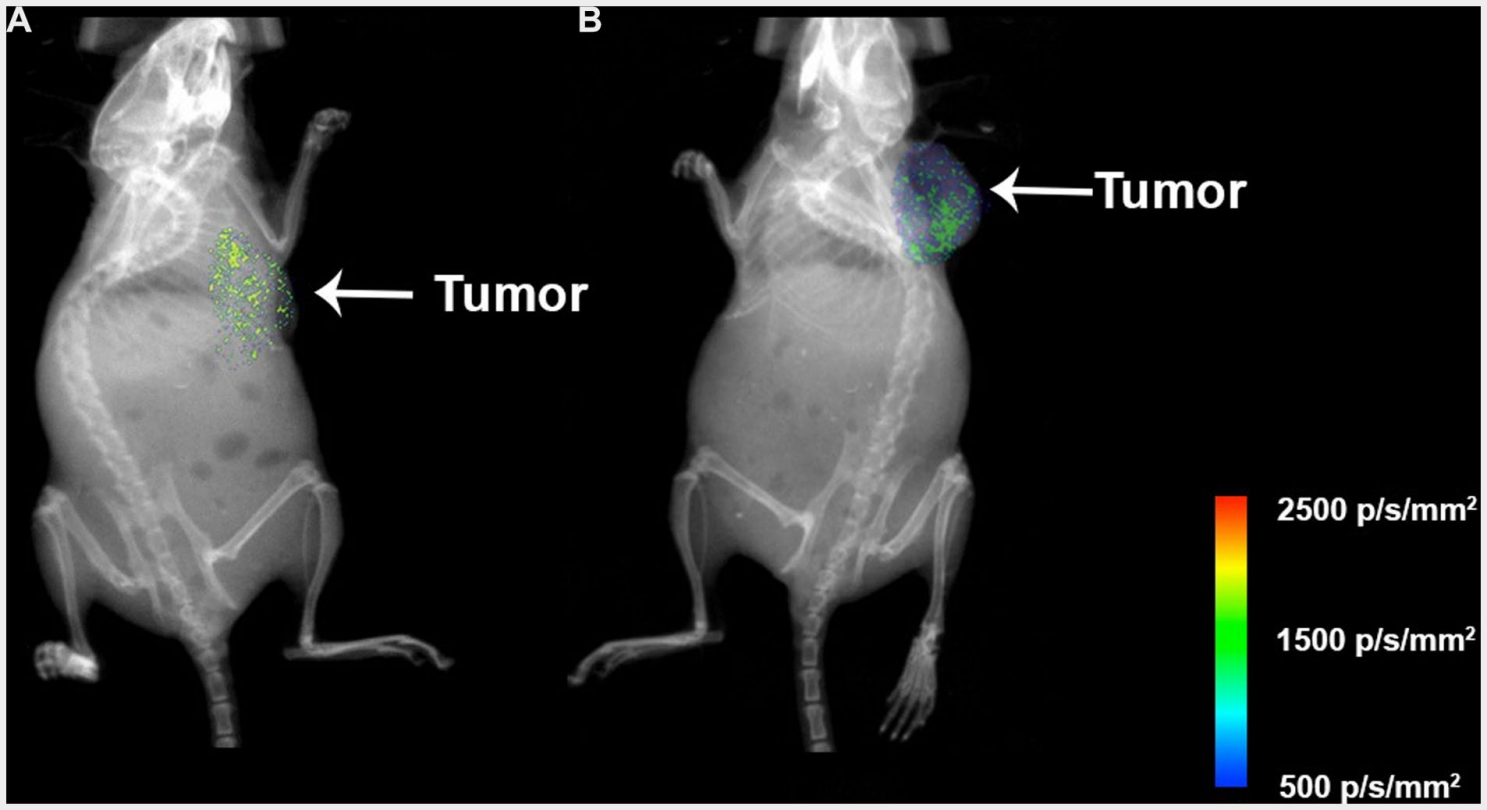
Xray and Radioisotopic images of mice treated with the **(A)** ^177^Lu-PLGA(RGF) system and **(B)** ^177^Lu-PLGA(RGF)-CXCR4L system 24 h post-administration of 2.4 MBq. The liver and kidney uptake was subtracted from the image to visualize the tumor uptake. Photons/s/mm^2^ (p/s/mm^2^).

The nanosystems and their respective controls were evaluated *in vivo* through colorectal cancer subcutaneous xenograft tumors in Nu/Nu mice. The mice were treated with ^177^Lu-PLGA(RGF)-CXCR4L, RGF (0.02 mg/mL), and ^177^Lu-PLGA(RGF). Furthermore, the combination of radiotherapy with ^177^Lu and chemotherapy with RGF lead to a significant decrease in relative tumor volume, compared with control (untreated) and free RGF (Figure 12A). Notably, mice treated with the final nanosystem showed a significant increase in survival rate (Figure 12B), while control and RGF mice succumbed on days 35 and 49, respectively. The proliferation of tumor cells through proliferating marker Ki67 was assessed to corroborate the effect of tumor volume reduction. Immunohistochemically, analysis of Ki67 expression showed that treatment with the nanosystem with or without CXCR4L reduces the proliferation of colorectal tumor cells. By contrast, Ki67 was significantly elevated in tumors without treatment (control; Figure 12C). Comparative radioisotopic images were taken of tumors in mice treated with nanosystems with and without CXCR4L after 24 h (Figures 13A,B). Both nanosystems display similar tumor uptake, which corroborates the highest inhibition of subcutaneous xenograft colorectal tumor with ^177^Lu-PLGA(RGF) and ^177^Lu-PLGA(RGF)-CXCR4L nanosystems.

Nanosystems have three retention mechanisms in healthy tissues, cells, and tumors. The first is phagocytosis through the reticuloendothelial system [nanoparticles uptake by liver macrophages (Kupffer cells)] and spleen macrophages. The second one is the fast accumulation in the tumor stroma (tumor microenvironment) through the enhanced permeability and retention (EPR) effect. The EPR effect initially takes advantage of the endothelial intercellular space size (from 400 nm up to 800 nm) of tumors (where nanoparticles easily cross and accumulate), compared to 2 nm in normal/healthy endothelial cells, where nanoparticles cannot cross. Once inside the tumor stroma, ^177^Lu-PLGA(RGF)-CXCR4L nanoparticles are also retained by the third mechanism, the specificity mediated by the CXCR4.

In summary, the first tumor interaction of ^177^Lu-PLGA(RGF)-CXCR4L through the EPR effect with high accumulation in the tumor stroma (but not in healthy tissues) and their speedy uptake by the reticuloendothelial system explains why other healthy tissues and immune cells expressing CXCR4 are not affected. ^177^Lu-nanoparticles safety for normal cells, using co-cultures of cancer cells and macrophages, histopathological evaluations, genotoxic assays, etc., has previously been demonstrated (45, 48).

**Figure 14:**
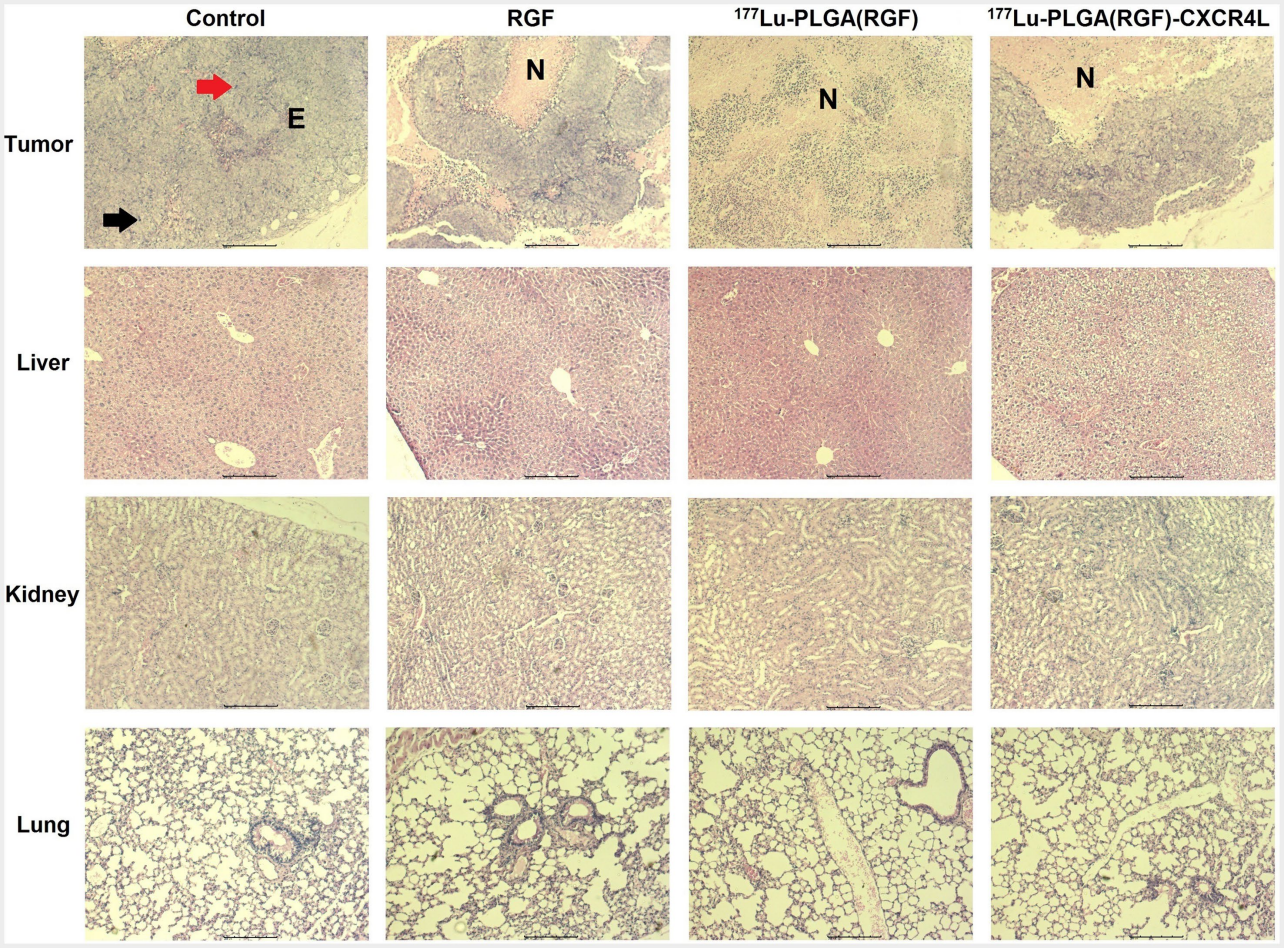
Microscopic overview of tumor, liver, kidney, and lung histopathology after 14 days of treatment of RGF, ^177^Lu-PLGA(RGF) and ^177^Lu-PLGA(RGF)-CXCR4L. The control is from untreated mice. Mitotic forms (red arrow); atypical nuclei (black arrow). Epithelial cells (E). Necrosis (N). The scale bar represents 200 μM.

One of the essential properties of nanoparticles as carriers of chemo-radiation therapy for cancer is validated mainly by its selective action toward tumors without damaging healthy organs (49). Micrographs show histological characteristics that define colorectal adenocarcinomas in tumors of the subcutaneous xenograft model of HCT116 cells, including epithelial cells and pleomorphic cell elements, mitotic forms (red arrow), and atypical nuclei (black arrow; Figure 14, upper panel). In addition, tumors treated with the ^177^Lu-PLGA(RGF)-CXCR4L system and with the ^177^Lu-PLGA(RGF) system had a necrotic area at the tumor center; inflammatory infiltration was not observed in tumor tissues, which would be an indication of apoptotic cell dead (50). In RGF-treated mice, necrotic areas were surrounded and reduced at the periphery of the tumor. Histological examination of the liver in mice treated with ^177^Lu-PLGA(RGF)-CXCR4L showed minor cytoplasmic degeneration, no steatosis, micro and macro vesicles, no necrotic foci. There was recruitment of Kupffer cells for nanoparticle endocytosis, but there was no identification of bleeding and infiltration of inflammatory cells. In some cases, binucleate cells were observed, indicating the regeneration process. Renal histopathological evaluation showed that exposure to ^177^Lu-PLGA(RGF)-CXR4L did not cause pathological changes, no diminished and distorted glomeruli were identified, no dilated tubules or edema exudate, neither necrosis nor infiltration of inflammatory cells was identified. Micrographs of the lung sections of the control group and the groups treated with RGF, ^177^Lu-PLGA(RGF) and ^177^Lu-PLGA(RGF)-CXCR4L showed a normal lung architecture with spongy structure, normal transparent alveoli, and thin interalveolar septa (Figure 14, middle and lower panel). Weng et al. (36) reported that mice treated with RGF at a concentration of 20 mg/kg/day for 14 days did not present severe toxicity measured by loss of body weight and did not present morphological differences in the liver histological study. Therefore, data suggest that intravenously-administered ^177^Lu-PLGA(RGF)-CXCR4L and ^177^Lu-PLGA(RGF) nanosystems do not lead to systemic toxicity or side effects.

## 4. Conclusion

The ^177^Lu-PLGA(RGF)-CXCR4L nanoparticle system was designed, synthesized, and evaluated *in vitro* and *in vivo*, based on the combination of specific chemotherapy provided by RGF and targeted radiotherapy, mediated by CXCR4L and ^177^Lu. The nanoparticle system showed a synergistic effect capable of reducing the viability and proliferation of the HCT116 colorectal cancer cell line and significantly decreasing tumor growth of the colorectal cancer xenograft model. The preparation of ^177^Lu-PLGA(RGF)-CXCR4L from a lyophilized formulation makes this formulation a good candidate to be produced in a GMP-grade facility for clinical translation. The data obtained in this research justifies additional preclinical safety trials and clinical evaluation of ^177^Lu-PLGA(RGF)-CXCR4L nanosystem as a potential combined treatment of colorectal cancer. Further evaluation regarding the preclinical therapeutic efficacy of ^177^Lu-PLGA(RGF)-CXCR4L nanoparticles should be performed to demonstrate their clinical potential.

## Data availability statement

The original contributions presented in the study are included in the article/Supplementary material, further inquiries can be directed to the corresponding author.

## Ethics statement

The animal study was reviewed and approved by Institutional Animal Care and Use Committee of the National Institute of Nuclear Research (ININ).

## Author contributions

PC-N: conceptualization, formal analysis, investigation, methodology, and writing—review and editing. BG-B: data curation, formal analysis, validation, and visualization. AA-C: data curation, formal analysis, validation, visualization, and writing—review and editing. GR-N and CS-C: investigation, formal analysis, validation, and visualization. ML-G: supervision, visualization, and project administration. BO-G: conceptualization, formal analysis, funding acquisition, investigation, resources, visualization, and writing—review and editing. All authors contributed to the article and approved the submitted version.

## Funding

This study was supported by the Consejo Nacional de Ciencia y Tecnología (CONACyT, A1-S-38087) and was carried out as part of the activities of the “Laboratorio Nacional de Investigación y Desarrollo de Radiofármacos, ININ.”

## Conflict of interest

The authors declare that the research was conducted in the absence of any commercial or financial relationships that could be construed as a potential conflict of interest.

## Publisher’s note

All claims expressed in this article are solely those of the authors and do not necessarily represent those of their affiliated organizations, or those of the publisher, the editors and the reviewers. Any product that may be evaluated in this article, or claim that may be made by its manufacturer, is not guaranteed or endorsed by the publisher.
