# Supplementary material for: Chemo-radiotherapy with 177Lu-PLGA(RGF)-CXCR4L for the targeted treatment of colorectal cancer
Source: Front Med (Lausanne). 2023 Jun 12;10:1191315. doi: 10.3389/fmed.2023.1191315 (PMC10292846; doi:10.3389/fmed.2023.1191315)
Supplement: Supplementary file 1 [file Data_Sheet_1.pdf]

Chemo-radiotherapy with ^{177}Lu-PLGA(RGF)-CXCR4L for the targeted treatment of colorectal cancer

Pedro Cruz-Nova[1], Brenda Gibbens-Bandala[1]*, Alejandra Ancira-Cortez[1], Gerardo Ramírez-Nava[2], Clara Santos-Cuevas[1], Myrna Luna-Gutiérrez[1], Blanca Ocampo-García[1]*.

Supplementary Material

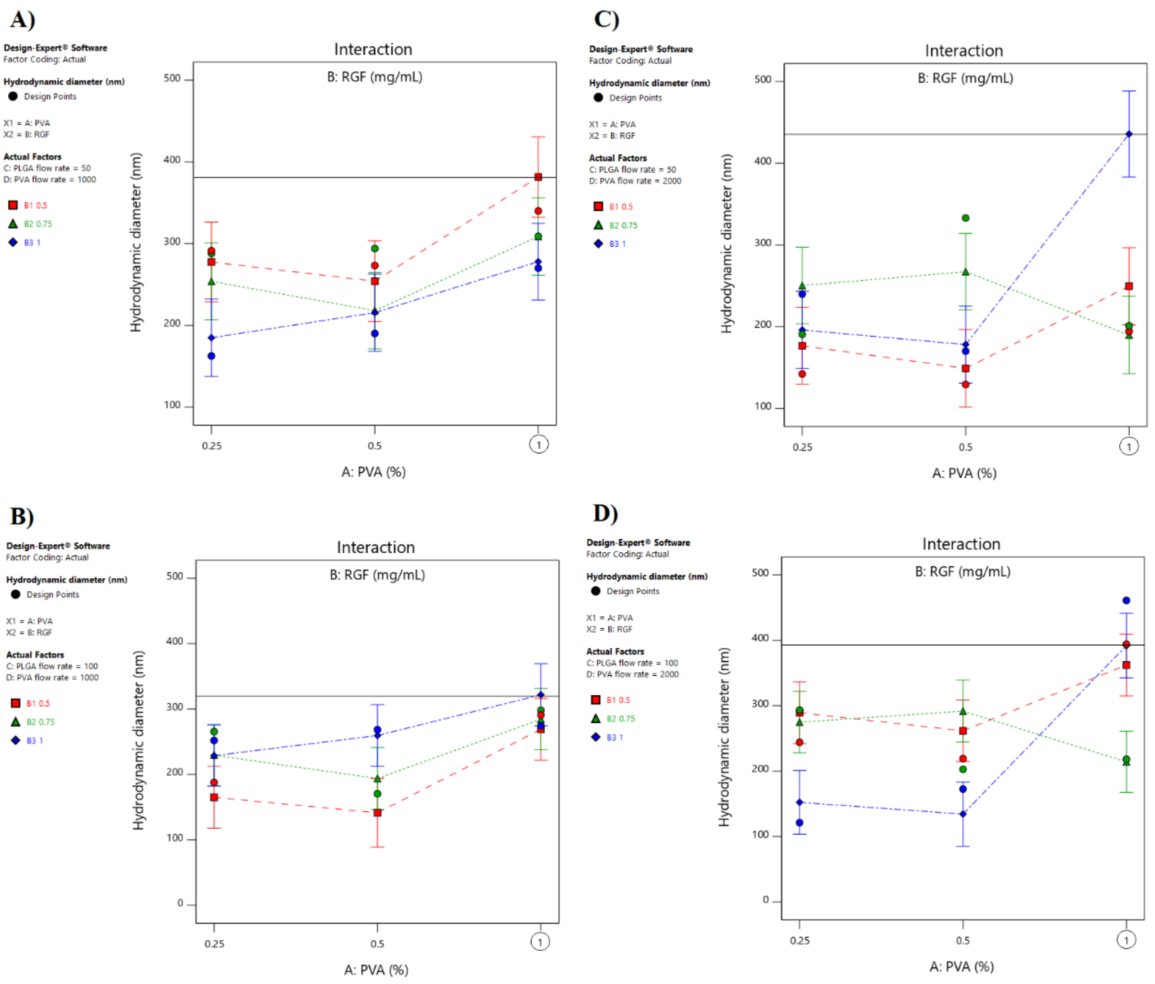

S1: Effect of PVA and RGF concentration and PLGA, PVA flow rate on the hydrodynamic diameter of PLGA(RGF) nanoparticles

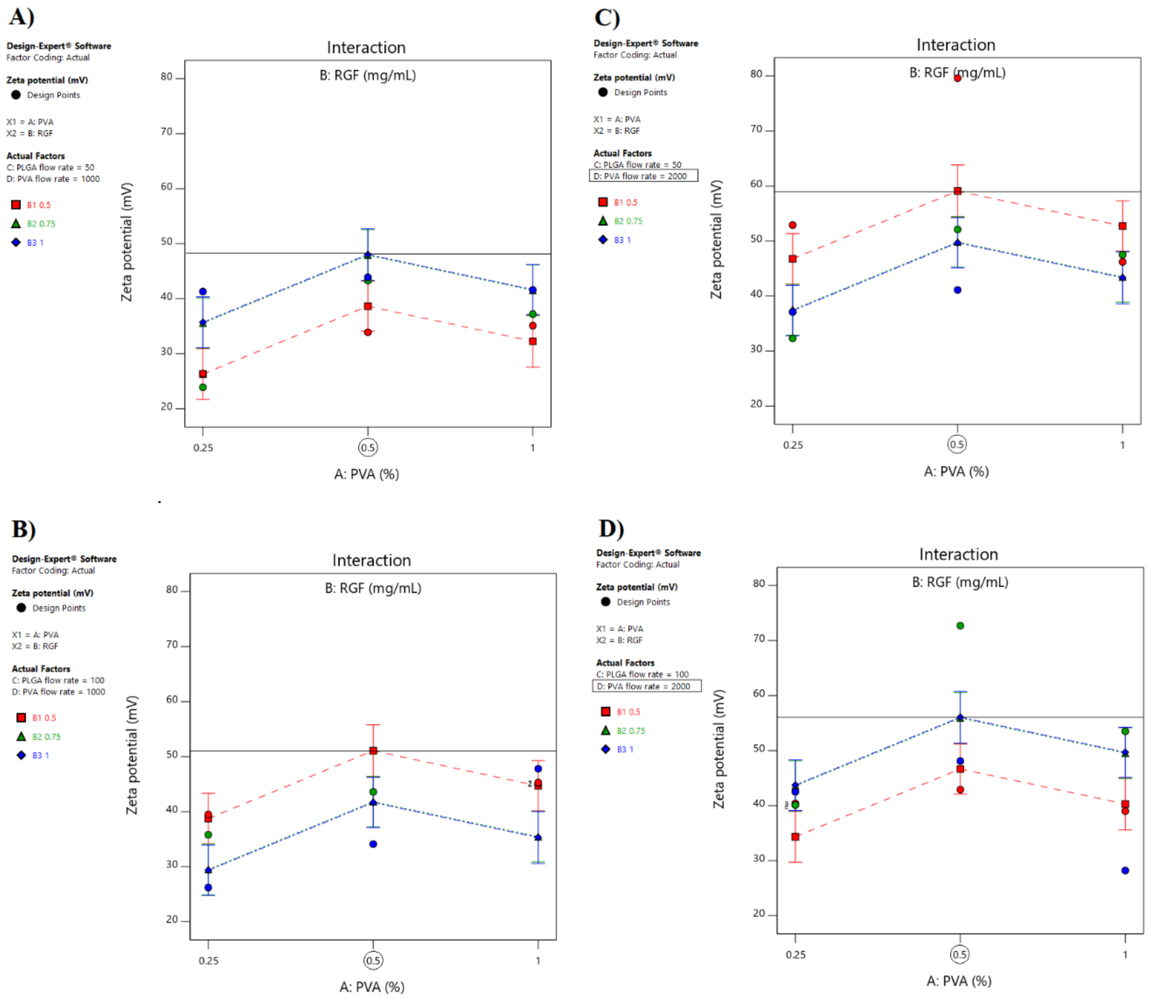

S2: Effect of PVA and RGF concentration and PLGA, PVA flow rate on the ζ potential of PLGA(RGF) nanoparticles

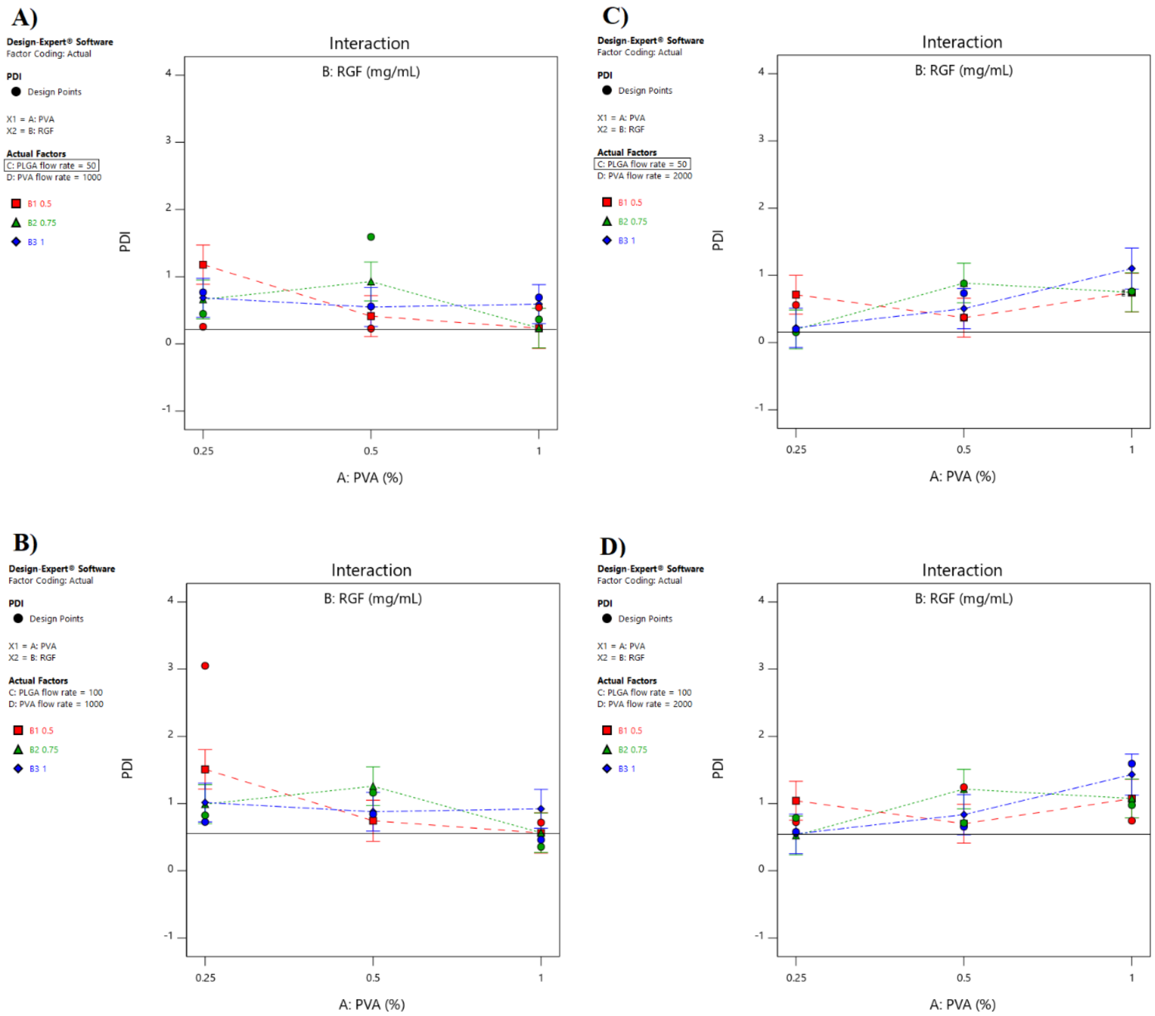

S3: Effect of PVA and RGF concentration and PLGA, PVA flow rate on the PDI of PLGA(RGF) nanoparticles

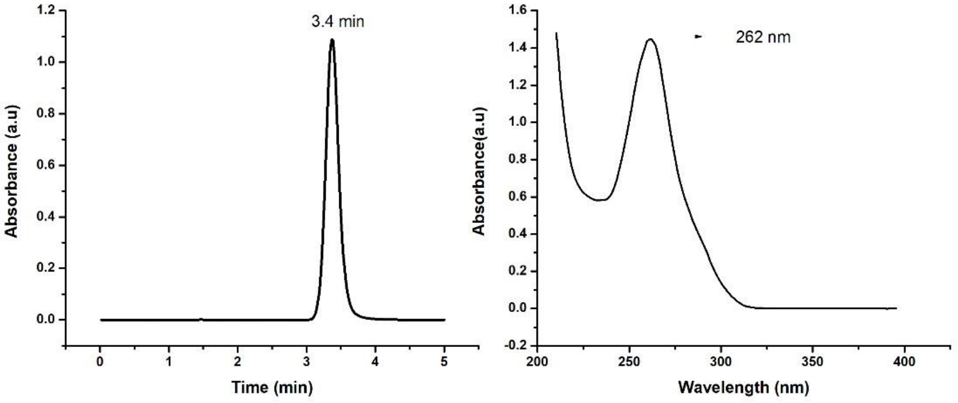

S4: Regorafenib chromatogram and the UV-Vis associated spectrum

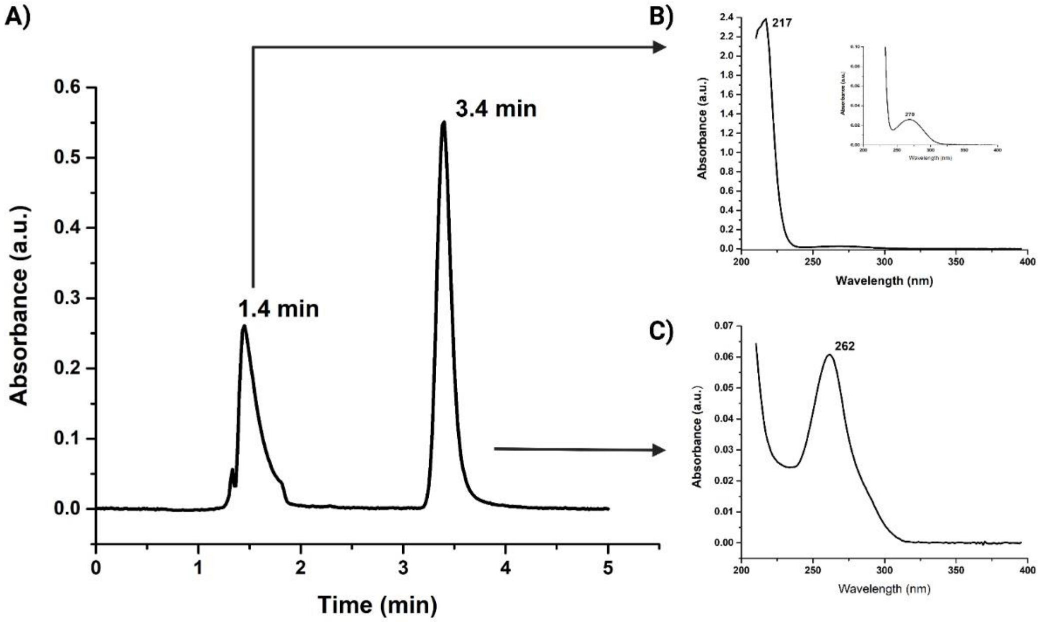

S5: A) Chromatogram of the recovered RGF from PLGA(RGF) nanoparticles. The peak at 1.4 nm corresponds to the UV-Vis spectrum of PLGA fragments (B) and the peak at 3.4 nm corresponds to recovered regorafenib (C). Dilution factor was not applied in B) and C)
